# Reversible, Polymeric Complexation of Therapeutic Peptides Using Esterification

**DOI:** 10.1021/acsmacrolett.6c00098

**Published:** 2026-03-30

**Authors:** Aditi S. Gourishankar, Mark S. Bannon, Kelly M. Bukovic, Earl Ashcraft, Laura M. Pfitzer, Rachel A. Letteri

**Affiliations:** † Department of Chemical Engineering, 118719 University of Virginia, Charlottesville, Virginia 22903, United States; ‡ Department of Chemistry, University of Virginia, Charlottesville, Virginia 22903, United States

## Abstract

Polyelectrolyte complexes permit the tunable, aqueous complexation of therapeutic peptides; however, their reliance on electrostatic and hydrophobic interactions limits complexation with weakly charged, hydrophilic peptides. While the peptide charge and hydrophobicity can be modified to encourage complexation, permanent modifications can hinder peptide activity and eventual release. Here, we show that esterifying the therapeutic peptide α-carboxyl terminus 11 (αCT11) reversibly increases its net charge and hydrophobicity, enabling complexation with anionic poly­(methacrylic acid) and subsequent dissociation, as esters hydrolyze over 24 h. Prior to hydrolysis, hydrophobic aspartimide intermediates appear to promote aggregation, prolonging complexation. Further emphasizing the importance of hydrophobicity, replacing the aromatic polymer chain end with a less hydrophobic group abrogated complexation. Together, these studies showcase how esterification can be leveraged for reversible polymeric complexation of weakly charged, hydrophilic peptides and present exciting prospects for using polymer hydrophobicity to tune reversible complexation to meet patient and delivery method-specific needs.

## Graphical abstract

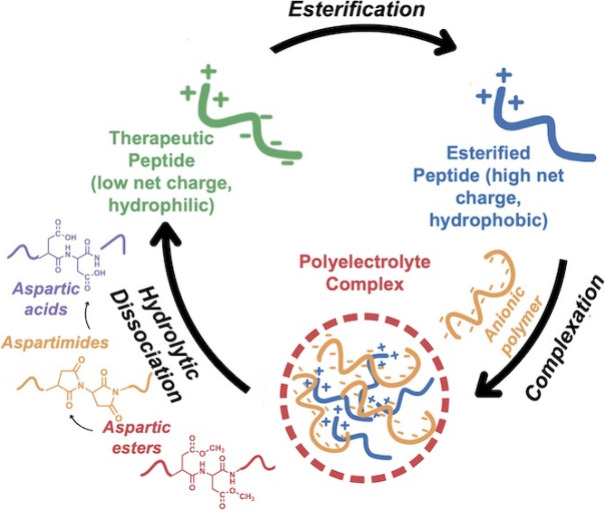

Therapeutic peptides combine the facile synthesis, storage, and transport of small-molecule therapeutics with the endogenous nature and activity of biologics.^1^ These advantages have led to commercially available peptide therapeutics, including insulin (regulates blood sugar),^2^ FUZEON (enfuvirtide, HIV-1 fusion inhibition),^3^ Oxytocin (labor induction and postpartum hemorrhage control),^4^ and most recently Ozempic (semaglutide, long-term weight management).^5^ Despite their promise, peptide therapeutics face multiple pharmacologic barriers, namely, enzymatic degradation and premature renal filtration, that shorten their lifetime and limit their clinical potential.^1,6–9^

To prolong lifetime, delivery vehicles can make peptide therapeutics sterically less accessible to enzymes to slow degradation and increase delivery cargo size to hinder renal filtration.^10–15^ Delivery vehicles formed from polyelectrolyte complexation offer the ability to encapsulate peptides in aqueous environments to avoid denaturing biologic cargo.^9,16–21^ Further, strategic polymer design that leverages electrostatic interactions and hydrophobic assembly can achieve a wide range of tunability in these delivery vehicles.^22–25^ While ideal for highly charged,^26,27^ hydrophobic, or large therapeutics like proteins,^33^ polyelectrolytes often struggle to complex small, hydrophilic peptides with low net charge.

One strategy to promote polyelectrolyte complexation with a therapeutic is to modify its net charge and hydrophobicity. Examples of such modifications in proteins include adding charged residues or modifying amino acid side chains to install charged groups.^28–34^ However, permanently modifying even a couple of amino acids can change the structure and detract from activity,^33^ particularly for smaller biologics such as peptides. Furthermore, permanently increasing the net charge on a peptide may cause the therapeutic to bind too strongly to a polymer, hindering its release to the target destination.^35,36^

Despite the challenges associated with complexation of weakly charged hydrophilic peptides, their promise warrants finding a solution. For example, the cardioprotective and wound-healing 9-amino acid α-carboxyl terminus 11 (αCT11, RPRPDDLEI) contains only 2 hydrophobic residues and a net charge of −1. While the inability to complex hinders αCT11 performance *in vivo*,^37^ up to 62% left ventricle recovery post ischemia reperfusion injury was achieved in *ex vivo* mouse models, illustrating its immense therapeutic potential.^38^ To realize efficient complexation without detracting from activity, reversible mechanisms to modify the net charge and hydrophobicity of therapeutic peptides such as αCT11 are required.

We previously showed that multisite esterification is a reversible modification strategy that replaces the 4 carboxylic acid groups on αCT11 with neutral, hydrophobic ester groups to increase its hydrophobicity and net charge from −1 to +3, which improved wound healing activity likely due to higher peptide cell membrane permeability.^39^ Here, we leverage peptide esterification to enable reversible complexation of αCT11 with an anionic polymer, poly­(methacrylic acid) (PMAA). We investigate the role of salt, peptide, polymer concentration, polymer chain length, and buffer type, as well as two potential side reactions. We found hydrophobicity to be a key variable in tuning the complex stability and dissociation time scales. Offering a reversible increase in peptide net charge and hydrophobicity, peptide esterification promotes the reversible complexation of weakly charged, hydrophilic peptides with polymers as a way to prolong their useful lifetime and elevate their clinical potential.

## Monitoring Time-Dependent Complexation of Esterified Peptide

To assess time-dependent complexation, we compared the turbidity of charge-balanced mixtures of the fully esterified peptide (αCT11-4OMe, Figures S4 and S5) and polymer (0.5 mM PMAA, DP = 23, Figure S1) to the unesterified peptide and polymer in potassium phosphate buffer (pH = 7.35–7.4, Figure S39) over 20 h. The esterified peptide and polymer mixture starts with a turbidity ∼4 times higher, consistent with complexation (Figures 1B and S12). The high turbidity between 0–4 h drops over 4.5–10 h, suggesting subsequent dissociation. Notably, even at 20 h, the turbidity does not decrease to the level of the presumably uncomplexed unesterified peptide and polymer mixture. This indicates that while complexation is reversible, dissociation is not yet complete at 20 h. While the absolute turbidity of the replicates varied, this is typical for complexes that exhibit a distribution of particle sizes.^40^ Differences in shear during mixing may also contribute to this variation. Importantly, all 3 replicates display the same overall trend: an initially high turbidity followed by subsequent decrease that does not return to baseline over 20 h.

**Figure 1:**
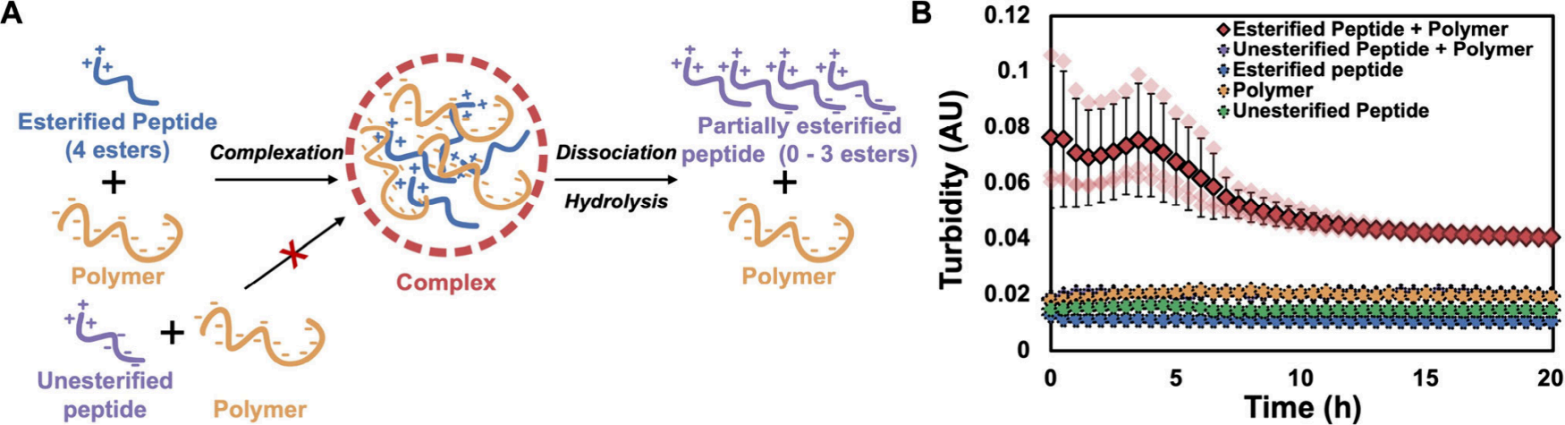
Effects of peptide esterification on complexation with an anionic polymer in 38 mM potassium phosphate buffer (pH = 7.35–7.4): (A) Schematic and (B) Turbidimetry of esterified peptide (αCT11-4OMe) + anionic polymer (PMAA) samples (average in red, *n* = 3 replicates in pink). The increase in turbidity followed by subsequent decrease in turbidity, when compared to controls of unesterified peptide + polymer (purple), esterified peptide (blue), polymer (yellow), and unesterified peptide (green) over 20 h, are consistent with complexation and subsequent dissociation of esterified peptide.

Microscopy corroborated the turbidity results. Optical microscopy showed large, irregularly shaped particles ranging from 5 to 75 μm only in the esterified peptide and polymer mixture (Figures S10 and S11). These particles appear to reduce in number by 20 h , but they do not completely disappear, consistent with incomplete dissociation. Transmission electron microscopy (TEM) showed an interconnected nanoscale mesh-like morphology initially (*t* = 0 h) that disappears by 22 h (Figures S8 and S9), consistent with the esterified peptide reversibly assembling with polymer, causing turbidity.

To rule out peptide or polymer aggregation contributing to turbidity, we show control measurements on solutions of the individual esterified peptide, unesterified peptide, and polymer (Figure 1B). These controls all showed consistently low turbidity, similar to the unesterified peptide and polymer mixture. Then, to ensure that turbidity decrease between 4.5 and 10 h was not due to gravitational settling of the complexes, we show that resuspending the complexes via pipetting before each measurement yielded the same turbidity increase and subsequent decrease (Figure S13).

Despite expecting a monotonic decrease in turbidity due to hydrolysis-induced dissociation, we see a highly reproducible peak in turbidity around 4.5 h (Figures 1B, S13, and S16–S18). To investigate this nonmonotonic turbidity, we monitored esterified peptide hydrolysis using liquid chromatography quadrupole time-of-flight (LC-QTOF) mass spectrometry (Figures 2B and S29–S33) and reverse-phase analytical high-performance liquid chromatography (RP-HPLC) (Figure S22). The extracted ion chromatogram (EIC) intensity gauges the abundance of a particular ion/species, while the elution time is proportional to the species hydrophobicity (Table 1). We calculate the abundance of a particular species as a percentage of the total peak area of the peptide-associated species.

**Figure 2:**
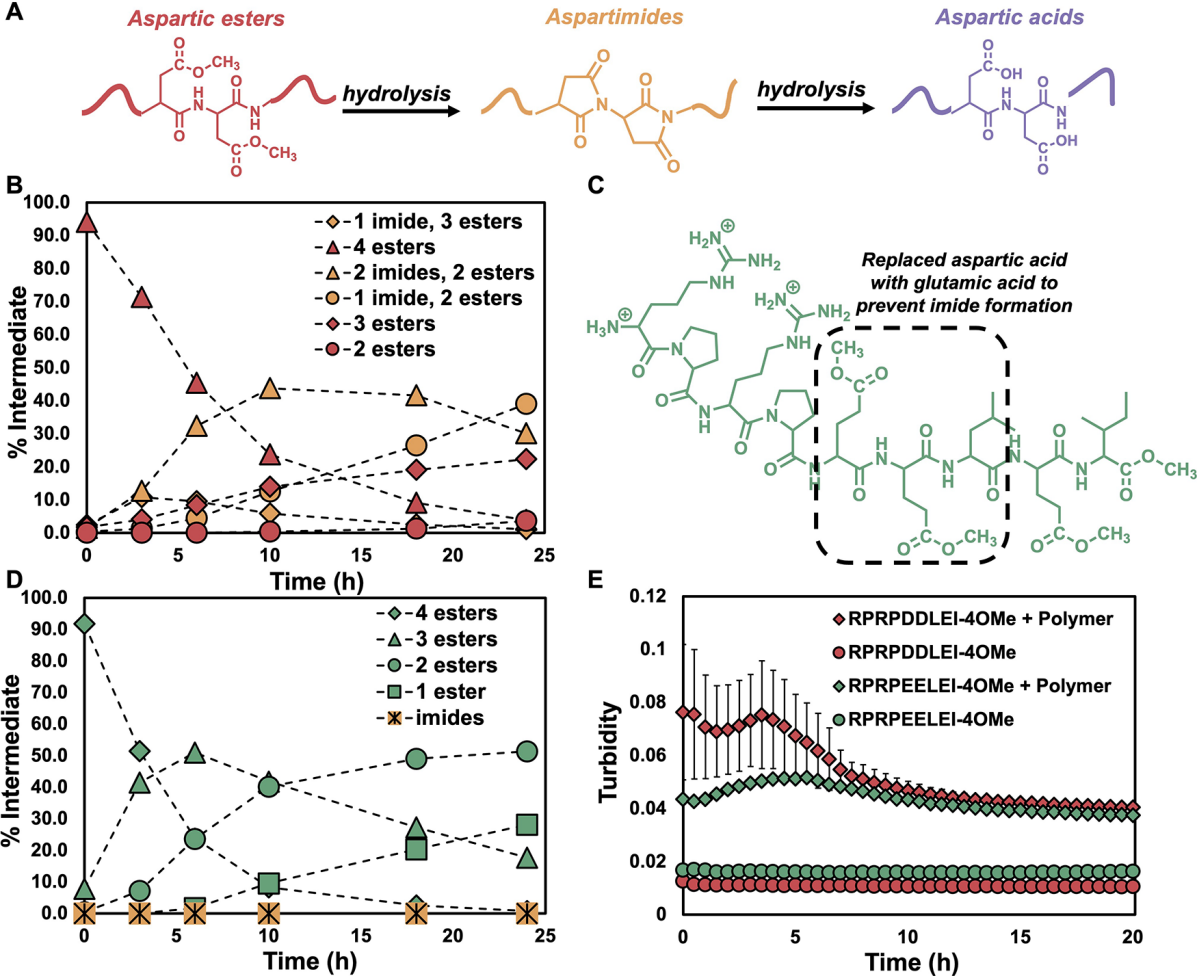
Assessing aspartimide formation in esterified peptide + polymer complexes: (A) Schematic of aspartimide formation (yellow) from aspartic esters (pink), ultimately reverting to aspartic acids (purple); (B) Tracking the % intermediates (peptide containing 2–4 esters and 1–2 aspartimides); and isolating effects of aspartimides using a control peptide – RPRPEELEI-4OMe, structure shown in (C) that cannot form aspartimides; (D) % Intermediates (esters and aspartimides) showing no imide formation in RPRPEELEI-4OMe over 24 h, under base-catalyzed conditions; and (E) Turbidity traces of RPRPDDLEI-4OMe + polymer (pink, diamond) in physiological conditions and RPRPEELEI-4OMe + polymer (green, diamond) in base-catalyzed conditions, to compare complexation and dissociation over 24 h. Controls of individual peptides are shown in circles.

**Table 1:** Retention Times of Hydrolysis Intermediates

species	retention time (min)
αCT11-3OMe-1imide	13.5
αCT11-4OMe	13.2
αCT11-2OMe-2imide	12.9
αCT11-2OMe-1imide	12.1
αCT11-3OMe	11.5

As expected, the abundance of the fully esterified peptide (αCT11–4OMe) decreased from 94 to 4% over 24 h. As αCT11-4OMe hydrolyzes, we see the appearance of intermediate-associated peaks, including peptide with 3 (αCT11-3OMe) and 2 esters (αCT11-2OMe) that each grow steadily to ∼20–25% abundance at 24 h. Interestingly, we also see the formation of a more hydrophobic species than the fully esterified peptide (αCT11-4OMe) at intermediate times, offering a possible explanation for the nonmonotonic turbidity. To identify this intermediate, we next considered two possible side reactions that may occur during hydrolysis: (1) imide formation on the esterified peptide and (2) aminolysis of the polymer end-group.

## Effects of Imide Formation on Complexation

During prior hydrolysis studies with the esterified peptide in the absence of polymer,^39^ we saw esters form imides before returning to acids (Figure 2A). Here, we evaluated imide formation during hydrolysis in the presence of polymer, again using LC-QTOF to simultaneously gauge the abundance and hydrophobicity of intermediate species.

LC-QTOF showed 3 imide species in the esterified peptide and polymer mixture: (1) αCT11-2OMe-1imide (2 esters, 1 imide, 12.1 min elution time), (2) αCT11-2OMe-2imide (2 esters, 2 imides, 12.9 min elution time), and, notably, the most hydrophobic (3) αCT11-3OMe-1imide (3 esters, 1 imide, 13.5 min elution time) (Figure 2B). The most hydrophobic species (αCT11-3OMe-1imide) reached its highest abundance between 2 and 6 h, before dropping to ∼1% abundance by 24 h, while the most hydrophilic (αCT11-2OMe-1imide) of these continued to form over 24 h, reaching 40% abundance. From the retention times (Figures S29–S33), we note that an imide imparts more hydrophobicity to the peptide than an ester, as the intermediate containing 2 esters and 1 imide (αCT11-2OMe-1imide) was more hydrophobic than that containing 3 esters (αCT11-3OMe). This trend extended to other imide intermediates, with the αCT11-3OMe-1imide being more hydrophobic than αCT11-4OMe. These data point to the combined hydrophobicity and abundance of the intermediates present between 2–6 h as a plausible explanation for the nonmonotonic turbidity that peaks during that time frame.

To further understand the contribution of imides to turbidity, since glutamic acids are less prone to imide formation,^41,42^ we replaced the aspartic acids on the fully esterified peptide (αCT11-4OMe, RPRPDDLEI-4OMe) with glutamic acids (RPRPEELEI-4OMe, Figures 2C, S6, and S7). Using LC-QTOF (Figures 2D and S34–S38), we confirmed that RPRPEELEI-4OMe does not form imides over 24 h, and thus, imide formation is confined to aspartic esters. Combining RPRPEELEI-4OMe with polymer in potassium phosphate buffer (pH 7.35–7.4) (Figures S20 and S21) showed turbidity indicative of complexation. Notably, this turbidity was 3 times higher than that of the RPRPDDLEI-4OMe and polymer mixture and did not drop over time. Consistently, reverse phase analytical high performance liquid chromatography (RP-HPLC) indicated that RPRPEELEI-4OMe is more hydrophobic than RPRPDDLEI-4OMe and did not hydrolyze over 24 h (Figure S25). Therefore, we compared the turbidity profiles of mixtures of RPRPDDLEI-4OMe and polymer to those of RPRPEELEI-4OMe and polymer under conditions where the two peptides hydrolyze similarly. Figure 2B shows the % RPRPDDLEI-4OMe to drop below 5% within 24 h in pH 7.35-7.4 potassium phosphate buffer, whereas RPRPEELEI-4OMe does the same at pH 10 (Figures 2C and S26). Thus, for the comparison, we monitored mixtures of RPRPDDLEI-4OMe and polymer in pH 7.35–7.4 potassium phosphate buffer and mixtures of RPRPEELEI-4OMe and polymer in pH 10 carbonate buffer (Figures S19 and S26). While less prominent than the RPRPDDLEI-4OMe complexes, the RPRPEELEI-4OMe complexes still showed some nonmonotonic behavior (Figure 2E). Considering the limitations of this comparison, including changing the buffer, pH and peptide, our strongest evidence suggesting that aspartimides at least partially contribute to the nonmonotonic turbidity between 2 and 6 h, remains the LC-QTOF data showing the highest abundance of the most hydrophobic aspartimide intermediate during the same timeframe. Together, these data suggest that aspartimides are an important consideration in assessing time dependent phase behavior in these systems.

## Effects of Aminolysis on Complexation

Another side reaction that could contribute to the nonmonotonic turbidity is aminolysis, in which the peptide N-terminal amine cleaves the polymer dithioester end group. This amine-reactive end group comes from the chain transfer agent (CTA) used to control the molecular weight in reversible addition–fragmentation chain transfer polymerization. Aminolysis would result in the peptide gaining the hydrophobic CTA end group, which could cause aggregation and thus increased turbidity.

First, to assess the effect of the CTA end group on turbidity, we replaced it with an isobutyronitrile end group (Figure S2), conferring resistance to aminolysis. We then combined the esterified peptide with the end-group-replaced (EGR) polymer and monitored the turbidity over 20 h. We saw low turbidity (Figure 3B), indicating no complexation. While this suggests that the hydrophobic CTA end group is important for complexation, two possibilities may explain this: (1) the CTA end group undergoes aminolysis and the hydrophobic product causes turbidity; or (2) the CTA end group on the polymer increases hydrophobic assembly with the esterified peptide.

**Figure 3:**
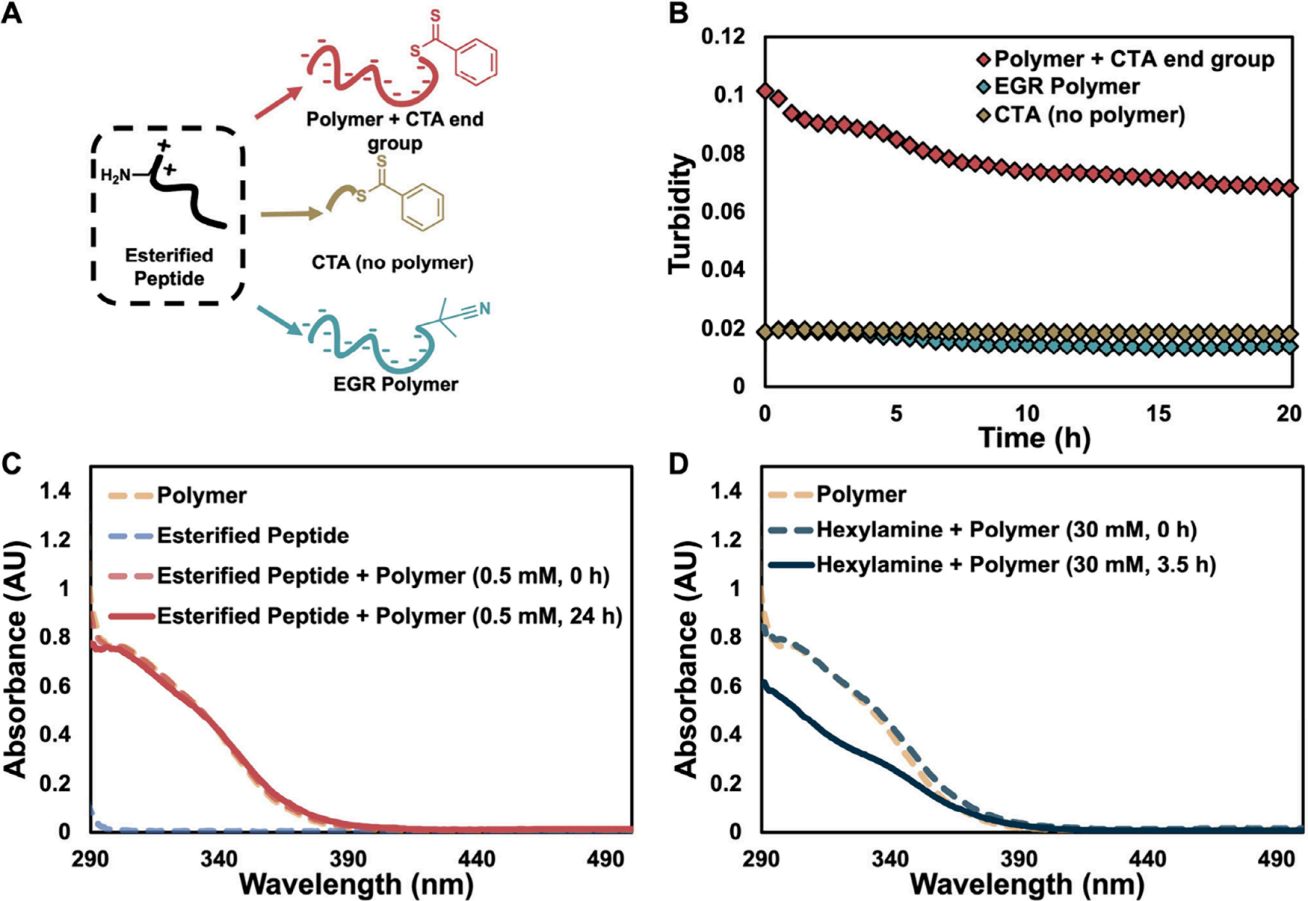
Isolating the effects of polymer end group (susceptible to aminolysis) on complexation: (A) Schematic of comparison groups, isolating effects of the polymer chain (yellow group) and the polymer end group (green group) on complexation; (B) Turbidity; (C) UV–visible spectrometry of polymer (end group signal ∼ 300 nm) in the presence (pink) and absence of (yellow) esterified peptide, over 24 h; (D) Positive control of aminolyzed polymer using excess hexylamine (blue). Dashed lines represent controls and solid lines represent samples; concentration reported represents polymer concentration prior to dilution for measurement.

We first performed a model reaction between the esterified peptide and the small molecule CTA containing the same amine-reactive dithioester. If aminolysis occurs and the hydrophobic peptide byproduct causes turbidity, we would expect an increase in turbidity upon reaction with the small molecule CTA as well. However, we saw no turbidity in the esterified peptide + small molecule CTA mixture (Figures 3B and S23). Further, aminolysis of the CTA end group caused a color change from pink to yellow,^43–45^ yet we saw no substantial color change visually or by UV–visible spectroscopy over 24 h (Figure 3C). As a positive control, we aminolyzed the CTA end group on the polymer using excess amounts of a smaller amine (hexylamine) and here observed a color change from pink to yellow, along with a reduction of absorbance at ∼300 nm via UV–visible spectroscopy (Figure 3D), after just 3.5 h. Together, these studies suggest that while aminolysis is possible under certain conditions, it does not occur under the conditions in which we conducted our turbidimetry experiments (3.3 mM amine, aqueous solvent). Therefore, it is more likely that the hydrophobic CTA end group on polymer is contributing to assembly.

## Effects of Solution Conditions and Polymer Length on Complexation

With hydrophobicity emerging as a key variable for tunability, we next assessed the role of solution conditions and polymer length on complex stability and the dissociation timeline. We first varied the salt concentration as well as the concentrations of peptide and polymer while maintaining the ratio of peptide:polymer. Doubling the concentration of the potassium phosphate in the buffer (pH = 7.35–7.4) from 38 mM to 76 mM disables complexation (Figures S15 and S24), confirming the expected role of electrostatic interactions. Complexation also depends on peptide and polymer concentration, where increasing the peptide and polymer concentration 6× (from 0.75 to 0.125 mM) increases the turbidity by 6× (from 0.02 to 0.12 AU) (Figure S16). Upon increasing polymer length, PMAA32 (DP = 32, Figure S3) complexed similarly to PMAA23 (DP = 23) with esterified peptide, despite having fewer hydrophobic end-groups (Figure S18), suggesting the importance of polymer chain length for complexation in these systems.

Varying the buffer type, we repeated experiments with the zwitterionic HEPES (4-(2-hydroxyethyl)-1-piperazineethanesulfonic acid) buffer and MOPS (3-(*N*-morpholino)­propanesulfonic acid) buffer, maintaining the 38 mM salt concentration and pH (7.35–7.4). The complexes formed in HEPES showed over 3× the turbidity of those in potassium phosphate buffer (Figure S14). Further, this turbidity did not drop to that of the unesterified peptide/polymer mixture by 24 h. Consistently, RP-HPLC shows the complexes hydrolyze much slower in HEPES buffer (Figure S28). In MOPS, the turbidity lies between that in potassium phosphate buffer and HEPES buffer (Figure S27). As expected, RP-HPLC suggests the complex hydrolysis in MOPS is slower than in potassium phosphate but higher than HEPES. Together, these data emphasize that tuning even just the type of buffer can tune hydrolytic dissociation time scales and complex stability in these systems.

## Conclusions

Through this work, we demonstrate a strategy to reversibly complex αCT11 under aqueous conditions, enabling its delivery while protecting it from enzymatic degradation and renal filtration to extend its therapeutic half-life. Using esterification, we reversibly increase the net charge and hydrophobicity of the weakly charged, hydrophilic therapeutic peptide αCT11, and show that this modification enables reversible complexation with the anionic PMAA. We present various opportunities to tune the complex stability and hydrolytic dissociation time scales in this system, by varying salt, peptide and polymer concentration, polymer chain length, and buffer type. Notably, in switching from the aspartic acids to glutamic acids, we increase complex stability, but detract from the reversibility aspartic acids offer. Excitingly, we see hydrophobicity play a major role in reversible complexation on multiple fronts. Replacing less hydrophobic amino acids (aspartic acids) with more hydrophobic ones (glutamic acids) and the formation of hydrophobic imide intermediates substantially increases complex stability. Further emphasizing the importance of hydrophobicity, replacing the aromatic hydrophobic polymer end group with a less hydrophobic one completely disables complexation, despite being just one of 20 units on the polymer. With hydrophobicity being a key variable, this system presents several opportunities to tune complex stability and dissociation, leveraging peptide and polymer design. This approach is likely generalizable to other weakly charged, hydrophilic peptides, addressing a key limitation of this class of therapeutics, short circulatory half-life, and ultimately offering the opportunity to enhance their efficacy.

## Supplementary Material



## Notes

Experimental details and further characterization including: NMR spectra, HPLC chromatograms, MALDI-TOF MS spectra, TEM and optical microscopy images, and LC-QTOF data (PDF)
