# Supplementary material for: Reversible, Polymeric Complexation of Therapeutic Peptides Using Esterification
Source: ACS Macro Lett. 2026 Mar 30;15(4):611–7. doi: 10.1021/acsmacrolett.6c00098 (PMC13104159; doi:10.1021/acsmacrolett.6c00098)
Supplement: Supplementary file 1 [file mz6c00098_si_001.pdf]

Reversible, polymeric complexation of therapeutic peptides using esterification

Aditi S. Gourishankar[1], Mark S. Bannon[1], Kelly M. Bukovic[1], Earl Ashcraft[2], Laura M. Pfitzer[2], Rachel A. Letteri[1]*

[1]Department of Chemical Engineering, University of Virginia, Charlottesville, VA 22903, USA

[2]Department of Chemistry, University of Virginia, Charlottesville, VA 22903, USA

*Corresponding author: Rachel A. Letteri (rl2qm@virginia.edu)

Table of Contents

Section S1. Instrumentation and Methods 2

Materials 2

Instrumentation 3

Methods 5

Synthesis of αCT11 5

Esterification of αCT11 5

Synthesis of poly(methacrylic acid) (PMAA) 6

End-group replacement of poly (methacrylic acid) (EGR) 6

Section S2. Supporting data 7

Material characterization 7

Nuclear magnetic resonance (NMR) spectroscopy of poly (methacrylic acid) (PMAA) 7

NMR spectroscopy of end-group-replaced PMAA 8

NMR spectroscopy of PMAA (DP = 32) 9

Charecterization of esterified RPRPDDLEI-4OMe 10

Charecterization of esterified RPRPEELEI 11

Visualizing structures during reversible complexation 12

Transmission electron microscopy (TEM) 12

Optical microscopy 15

Effects of gravitational settling on turbidity 18

Turbidity of esterified peptide + polymer (resuspended before measurement) 18

Effect of peptide, polymer and salt concentrations, buffer type and pH correction on turbidity 19
Turbidity of esterified peptide + polymer in MOPS and HEPES buffer 19
Turbidimetry of esterified peptide + polymer complexes in 2X buffer (77 mM) 21
Turbidimetry of esterified peptide + polymer complexes across different peptide-polymer concentrations 21
Turbidimetry of esterified peptide + polymer complexes without pH correction 23
Effects of aspartimide intermediates on turbidity 25
Tracking hydrolysis during reversible complexation 27
Analytical reverse-phase high-performance liquid-chromatography (RP-HPLC) of esterified peptide under different conditions 27
Liquid chromatography quadrupole time of flight (LC-QTOF) mass spectrometry of RPRPDDLEI-4OMe + polymer complexes 32
Liquid chromatography quadrupole time of flight (LC-QTOF) mass spectrometry of RPRPEELEI-4OMe + polymer complexes 37
Monitoring pH of esterified peptide + polymer and controls over 20 h 42

Section S1. Instrumentation and Methods

Materials

Fluorenylmethoxycarbonyl(Fmoc)-protected amino acids and 2-chlorotrityl chloride resin (0.6 mmol/g) were purchased from Advanced ChemTech (Louisville, Kentucky). Diisopropyl carbodiimide (DIC, 99.8%), N,N'-dimethylformamide (DMF, 99%), Oxyma Pure (99%), piperidine (99%), N,N-Diisopropylethylamine (DIPEA, 99%), trifluoroacetic acid (TFA, 99%), triisopropylsilane (TIPS, 98%), 2,2'-(ethylenedioxy)diethanethiol (DODT, 95%), diethyl ether (99%), methanol (MeOH, > 99%), acetonitrile (ACN, HPLC-grade, 99.9%), hydrochloric acid (HCl, 37% in H2O), deuterated dimethyl sulfoxide (DMSO-d6, 99.5%), sodium bicarbonate (99.7%), sodium carbonate (99.5%), potassium phosphate monobasic (99%), potassium phosphate dibasic (99%), methacrylic acid (MAA, stabilized with monomethyl ether hydroquinone (MEHQ), > 99%), 4-Cyano-4-(phenylcarbonothioylthio)pentanoic acid (CTA, >99%), 4-4'-azobis(4-cyanovaleric acid) (ACVA, ≥ 98.0%), and 2,2'-azoisobutyronitrile (AIBN, 98%) were purchased from Sigma Aldrich. Potassium iodide (KI) was purchased from VWR. Prepacked hydroquinone (HQ)

and monomethyl ether hydroquinone (MEHQ) inhibitor removal column was purchased from Scientific Polymer Products Inc. MAA was purified by passing through an inhibitor removal column immediately before addition. All other chemicals were used as received. Reverse-osmosis (RO) water was produced using an in-house purification system. Ultrapure water (18.2 MΩ cm) was obtained from a Thermo Scientific Smart2Pure water purification system.

Instrumentation

MALDI-ToF MS was conducted on a Shimadzu MALDI-8030 mass spectrometer (Shimadzu Scientific Instruments, inc., Columbia, MD) with an α-Cyano-4-hydroxycinnamic acid (CHCA) matrix.

Analytical RP-HPLC was conducted using a Waters Alliance e2695 RP-HPLC system (Waters Corporation, Milford, MA) using a Symmetry C18 column (4.6 x 75 mm, 3.5 μm particle size, 100 Å pore size). Samples prepared in potassium phosphate buffer (10 μL) were diluted with 0.7 mL of 5% ACN containing 0.1% TFA and injected on the column, where a linear gradient from 0% to 23% to 26% acetonitrile (ACN)/water containing 0.1% trifluoroacetic acid (TFA) over 22 min at a flow rate of 1 mL/min was used.

Preparative-scale RP-HPLC was conducted at 25.52 mL min^{-1} using a Waters Empower system (Waters Corporation, Milford, MA) to isolate the fully esterified peptide. The system was equipped with a XBridge® C18 column (30 x 150 mm, 5 μm beads) and a photodiode array detector (Waters 2489 UV/visible) that monitored UV absorbance at 214 nm. The samples were prepared at ~10 mg/mL and injected through the column where a linear gradient over 70 min from 0% to 23% to 26% acetonitrile (ACN)/water containing 0.1% trifluoroacetic acid (TFA) was used. Like fractions were combined and lyophilized.

Turbidimetry was conducted on an Infinite 200 PRO (Tecan Group Ltd.) multimode plate reader. Measurements were conducted over 24 h, with measurements taken every 30 min. The samples were placed in a 96-well plate, with measurements conducted at a wavelength of 550 nm, at a bandwidth of 9 nm, and 25 measurements per run.

UV-vis spectroscopy was conducted using a Shimadzu UVmini-1240, with a wavelength range from 190 – 1100 nm. To conduct UV-Visible spectroscopy, esterified peptide + polymer samples were prepared by combining 0.5 mM polymer with 3.32 mM esterified peptide in 38 mM potassium phosphate buffer, and hexyl amine + polymer samples were prepared by combining 30 mM polymer with an excess of hexylamine in DMSO. All samples were pH corrected to a pH of 7.35-7.4. For each time point, an aliquot of the sample was taken out and diluted in potassium phosphate buffer to reach a final polymer concentration of 100 µM, and 1 mL of the resulting solution pipetted into a cuvette to run UV-vis spectroscopy.

LC-QTOF was conducted on an Agilent G7104C LC system equipped with an Agilent G1958–65268 Dual AJS electrospray ionization source and Agilent 6545B QTOF mass spectrometer and Agilent Poroshell 120 Stable Bond Aqueous C18 column. Samples were prepared by combining 0.5 mM polymer with 3.32 mM esterified peptide and pH corrected to 7.35-7.4. Samples were diluted and run with an initial solvent composition of 95/5/0.1 water/acetonitrile (ACN)/formic acid, for 18 min at a flow rate of 0.4 ml/min at 60 ^{o}C. The ACN concentration was increased linearly to 20% over 15 min. The ACN was then increased to 100% at 15.01 min and held for 3 min to flush the column. A 3 min post-time was used between samples to re-equilibrate the column.

Optical microscopy was performed using a Leica DMi1 inverted microscope, in conjunction with the Leica LasX software for image acquisition, analysis, and control. The esterified peptide and polymer complex samples were pipetted into a 96-well plate at 200 µL per well and imaged at 20X and 40X magnification.

TEM images were obtained using an FEI Titan Transmission Electron Microscope. Carbon-film grids (5-6 nm) were discharged using a plasma activator for 30 s per grid at 50% power. Sample solution (1-3 µL) was then pipetted onto the grid and excess solution along the edges was blotted using a Kimwipe. After drying for 1 min, samples were stained by inverting the grids (sample side down) into 20 µL of 2% uranyl acetate for 1

min. The excess staining solution was blotted using a Kimwipe, and the grid was allowed to dry for 3 h. The prepared sample grids were imaged at 120 kV.

Methods

Synthesis of αCT11

αCT11 was synthesized using a CEM Liberty Blue automated microwave-assisted peptide synthesizer via Fmoc-solid phase peptide synthesis. Using KI (0.125 M in dimethylformamide (DMF)) and DIPEA (1 M in DMF), the amino acids were coupled to the 2-chlorotrityl chloride resin (0.6 mmol/g), mediated by diisopropyl carbodiimide (1 M in DMF) and oxyma pure (1 M in DMF) at 25 ºC. The Fmoc groups were deprotected using piperidine (20% v/v in DMF). To isolate the resultant peptide from the resin, a deprotection cocktail (trifluoroacetic acid, water, triisopropylsilane, and 2-2'-(ethylenedioxy)diethanethiol – 92.5/2.5/2.5/2.5 v/v) was introduced to the peptide-resin and stirred for 3 h at room temperature. Following this reaction, the peptide was filtered and precipitated into cold diethyl ether and centrifuged for 5 min at 2420 xg and 4 ºC, to obtain a peptide pellet. This centrifugation step was repeated upon reintroduction of diethyl ether to wash the peptide, and the resultant pellet was dried under vacuum to remove remaining solvent, then lyophilized. Finally, the peptide was purified using preparative-scale RP-HPLC and characterized using analytical RP-HPLC and MALDI-TOF-MS.

Esterification of αCT11

MeOH was added to αCT11 in a molar ratio of 5000:1 ([alcohol]:[carboxylic acid]) then the esterification reaction proceeded for 24 h in a 20 mL vial with 5% v/v 12 M HCl, maintained at 40 ºC. Following the reaction, the product solution was precipitated in diethyl ether and centrifuged at 4700 rpm for 5 min, followed by removal of the supernatant. This step was conducted 2 times, followed by drying the product under vacuum through a Schlenk line. The final product was then purified using preparative-scale RP-HPLC. The resultant fully esterified peptide was lyophilized and stored at a temperature of -20 ºC.

Synthesis of poly(methacrylic acid) (PMAA)

Poly(methacrylic acid) was synthesized via reversible-addition fragmentation-chain transfer (RAFT) polymerization. MAA (1.7 mL, 20 mmol), CTA (279.3 mg, 1 mmol), ACVA (28 mg, 0.1 mmol), and MeOH (8.3 mL, monomer concentration of 2.4 M) were added synchronously to a 20 mL scintillation vial. After reagents dissolved, the solution was deoxygenated via bubbling with $N_{2(g)}$ for 30 min, then the vial was placed in a silicone oil bath set to 60 ºC for 24 h. The reaction was quenched by opening the vial to air. The reaction solution was then purified via dialysis using a dialysis membrane with a molecular weight cutoff (MWCO) of 1 kDa in a 4 L glass beaker with 2 solvent changes of 40/60 MeOH/water, then 2 solvent changes against water, with solvent changes occurring at least 4 h apart. Purified polymer was lyophilized and stored at -20 ºC until use.

End-group replacement of poly (methacrylic acid) (EGR)

PMAA (100 mg, 0.043 mmol) was dissolved in MeOH at a concentration of 2.4 M in a 20 mL scintillation vial equipped with a stir bar, then AIBN (714 mg, 4.3 mmol) was added. Once dissolved, the solution was deoxygenated via bubbling with $N_{2(g)}$ for 30 min, then placed in a bead bath set to 60 ºC for 4 h. At the end of this period, the reaction was quenched by opening the vial to air. The solution was then purified via dialysis using a dialysis membrane with a molecular weight cutoff (MWCO) of 1 kDa in a 4 L glass beaker with 2 solvent changes of 40/60 MeOH/water, then 2 solvent changes against water, with solvent changes occurring at least 4 h apart. The solution was then vacuum filtered to remove any remaining AIBN, then lyophilized and stored at -20 ºC until use.

<u>Section S2. Supporting data</u>

Material characterization

<u>Nuclear magnetic resonance (NMR) spectroscopy of poly (methacrylic acid) (PMAA)</u>

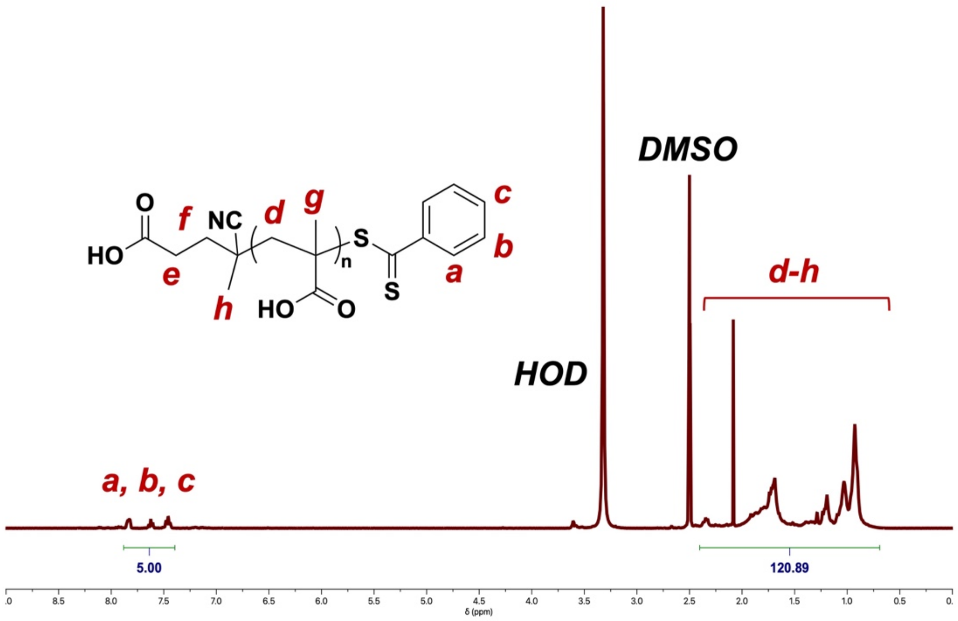

Figure S1. ^{1}H NMR (400 MHz, DMSO-d6) spectra of poly (methacrylic acid). The integration of the 5 phenyl protons (7.5 ppm, peak a, b, c) was set to 5 in the spectrum. Using end-group analysis, n was calculated to be 23.

NMR spectroscopy of end-group-replaced PMAA

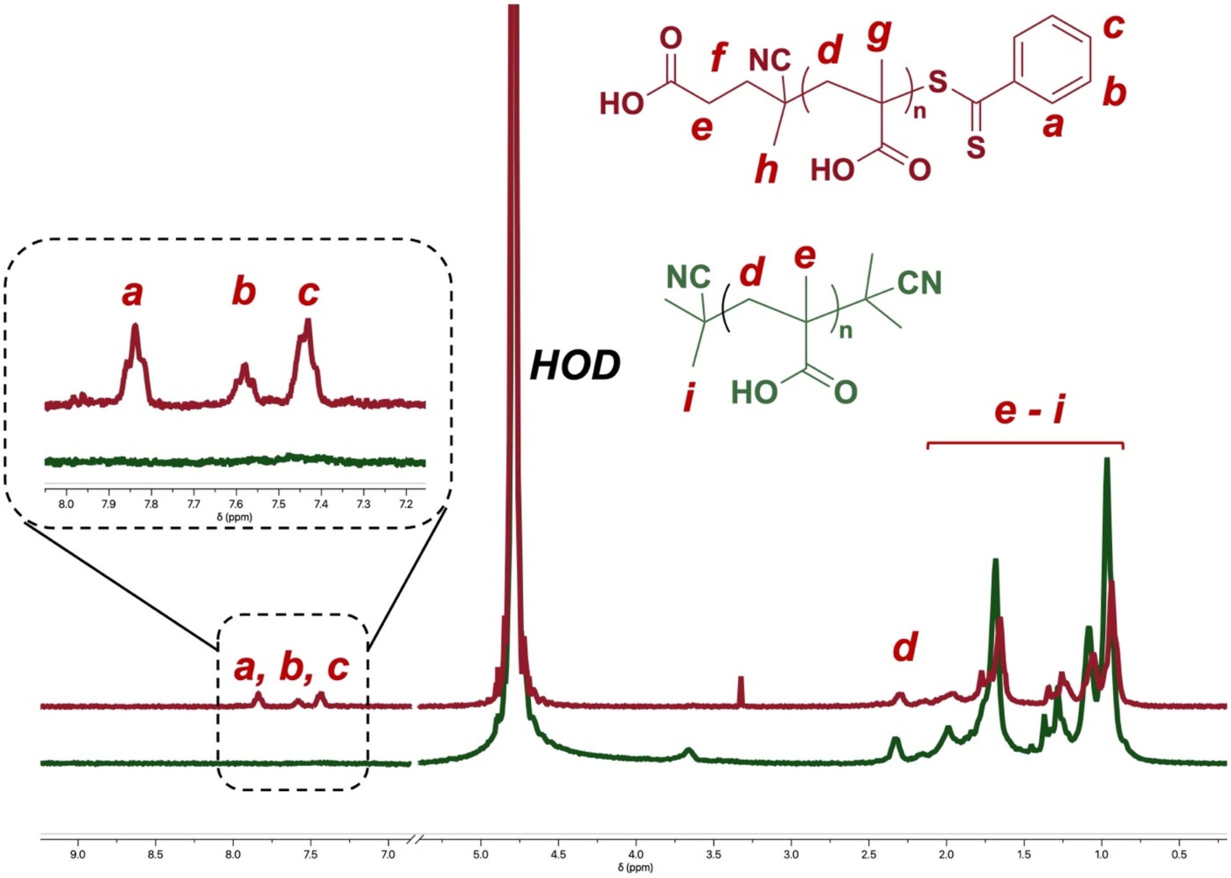

Figure S2. ^{1}H NMR (400 MHz) spectra of poly (methacrylic acid) (red) compared to end-group replaced poly (methacrylic acid) (green). The disappearance of the 5 phenyl protons (7.5 ppm, peak a, b, c) suggest a complete reaction.

NMR spectroscopy of PMAA (DP = 32)

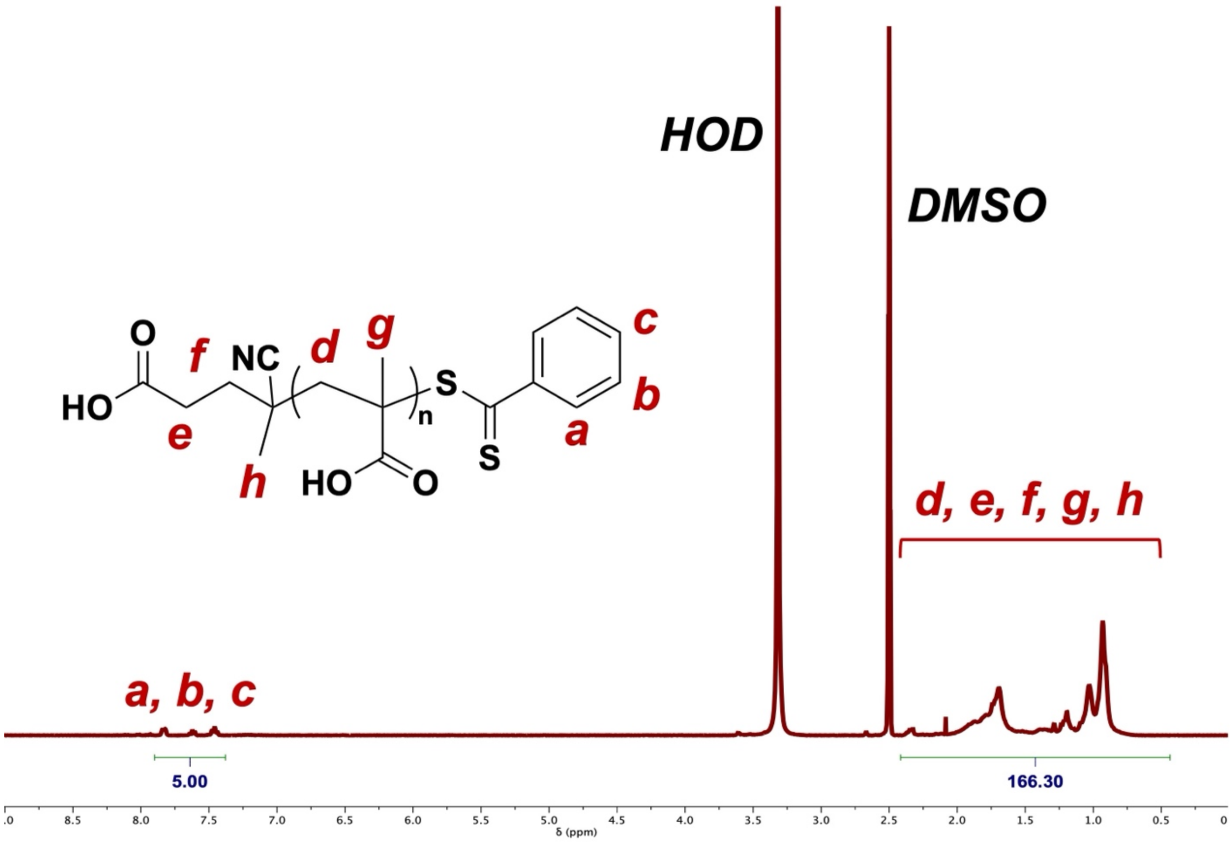

Figure S3. ^{1}H NMR (400 MHz, DMSO-d6) spectra of poly (methacrylic acid). The integration of the 5 phenyl protons (7.5 ppm, peak a, b, c) was set to 5 in the spectrum. Using end-group analysis, n was calculated to be 32.

Charecterization of esterified RPRPDDLEI-4OMe

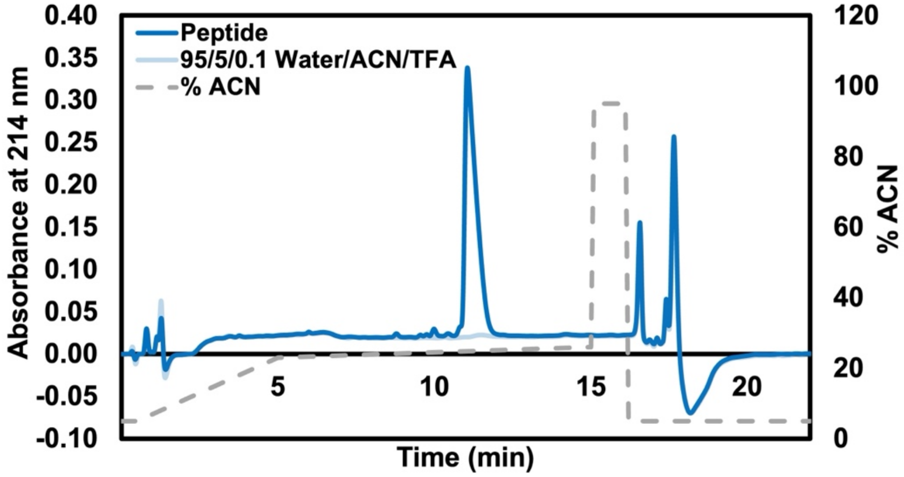

Figure S4. RP-HPLC chromatogram of esterified peptide (RPRPDDLEI-4OMe). Purity > 95% calculated using peak area.

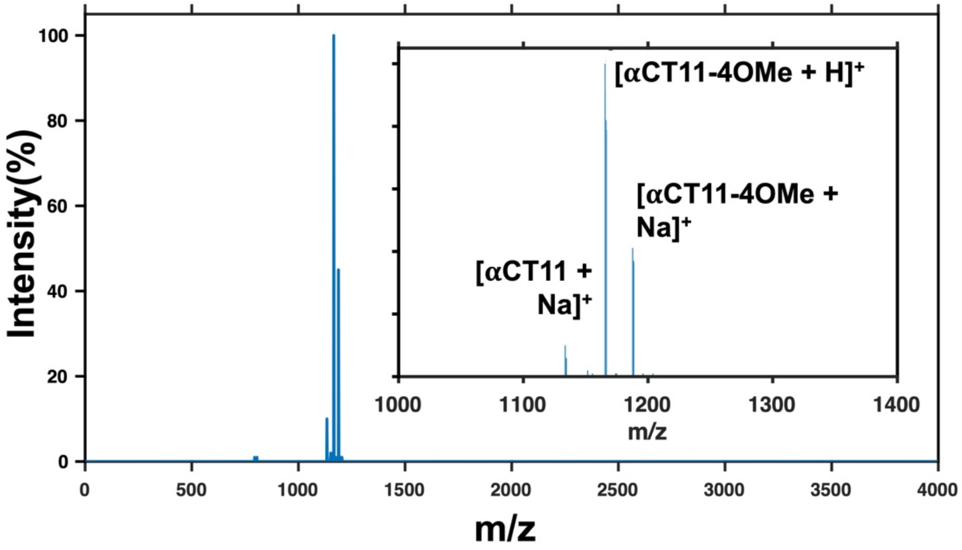

Figure S5. MALDI-TOF mass spectrometry of esterified peptide (RPRPDDLEI-4OMe)

Charecterization of esterified RPRPEELEI

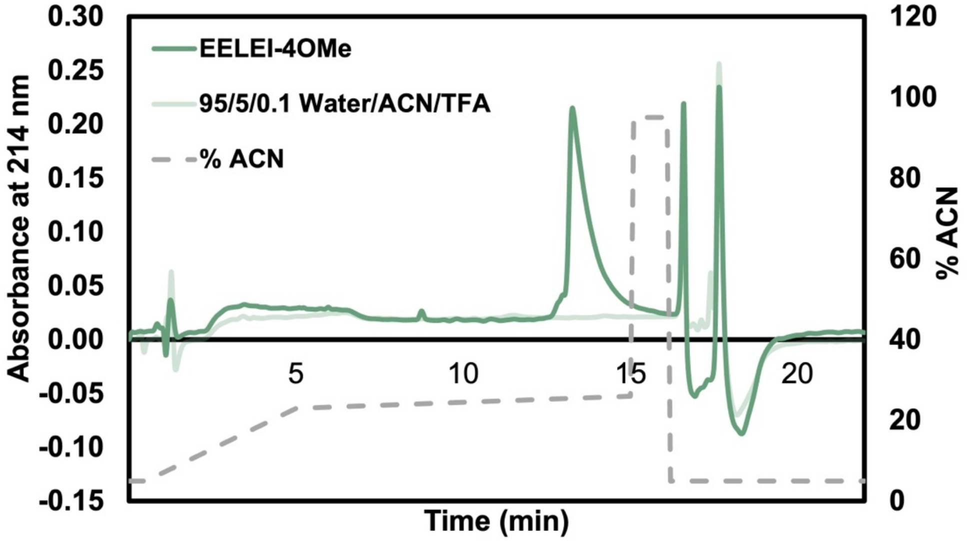

Figure S6. RP-HPLC chromatogram of esterified peptide (RPRPEELEI-4OMe), where aspartic acids have been replaced with glutamic acids, to prevent aspartimide formation. Purity >95% calculated from peak area.

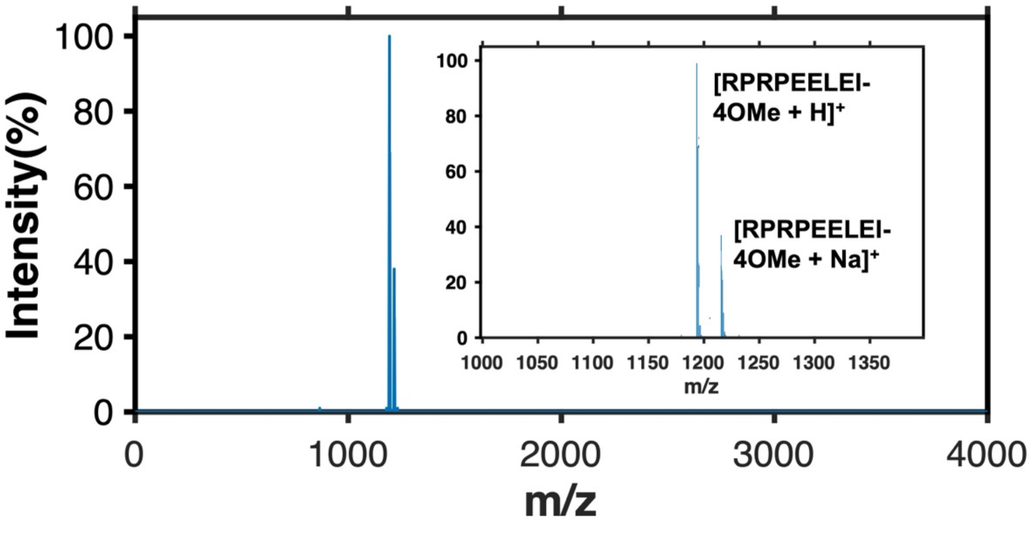

Figure S7. MALDI-TOF mass spectrometry of esterified peptide (RPRPEELEI-4OMe)

Visualizing structures during reversible complexation

Transmission electron microscopy (TEM)

We conducted TEM to visualize changes, over 22 h, to nanoscale structure of the blends of esterified peptide and polymer. Images of the esterified peptide + polymer mixture (**Figure S8**) were compared to controls of polymer, unesterified peptide, unesterified peptide + polymer, and esterified peptide (**Figure S9**), over 22 h. The esterified peptide + polymer forms a fibrous network-like structure initially (0 h time point), consistent with the initial high turbidity of these blends. This network structure disappears by 22 h, consistent with the drop in turbidity over that timeframe. No similar network structure was observed in the any of the controls.

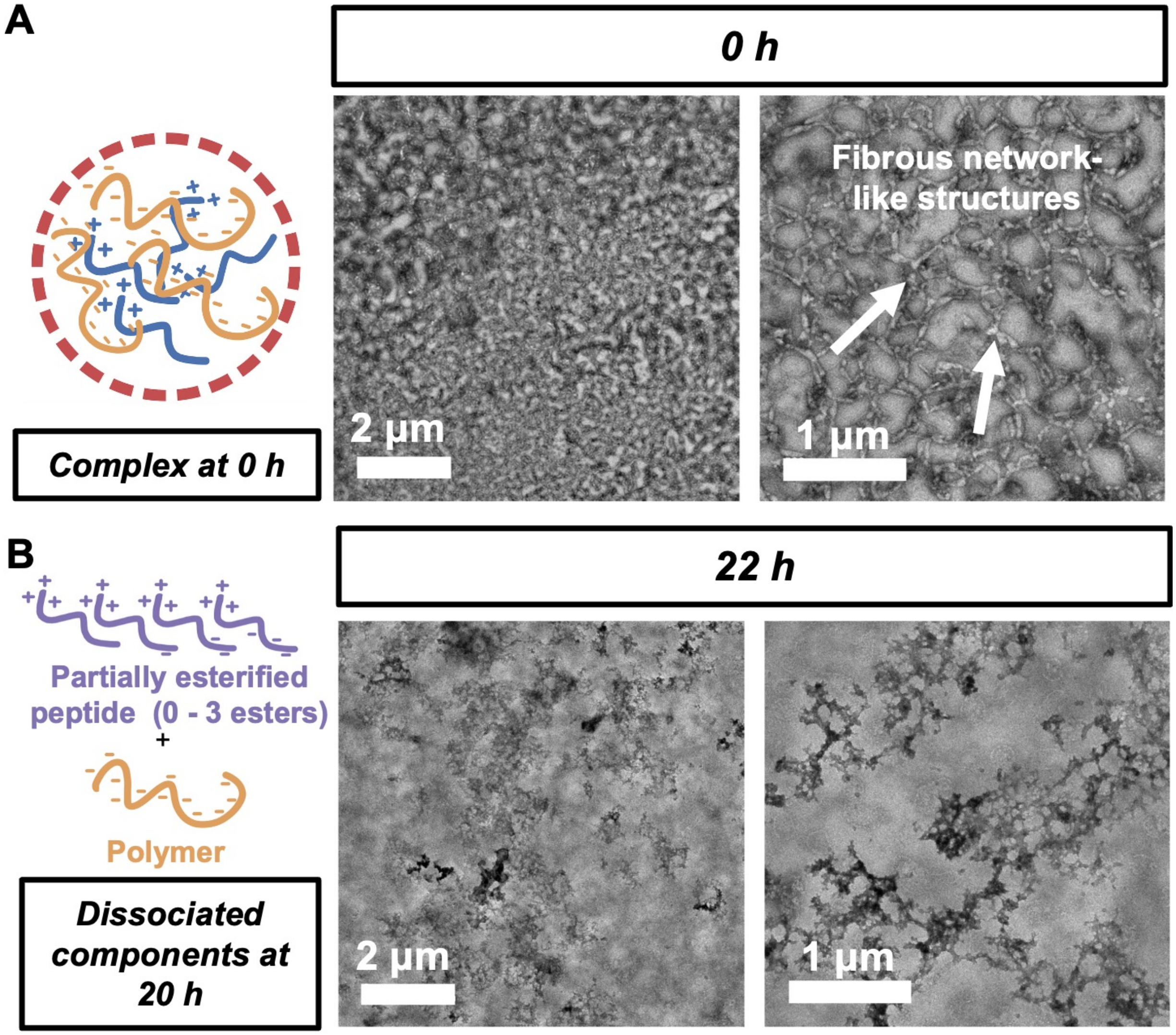

Figure S8. TEM of esterified peptide + polymer mixture after: A) 0 h; and B) 22 h of incubation in 38 mM potassium phosphate buffer, showing the fibrous network structures present initially to dissolve.

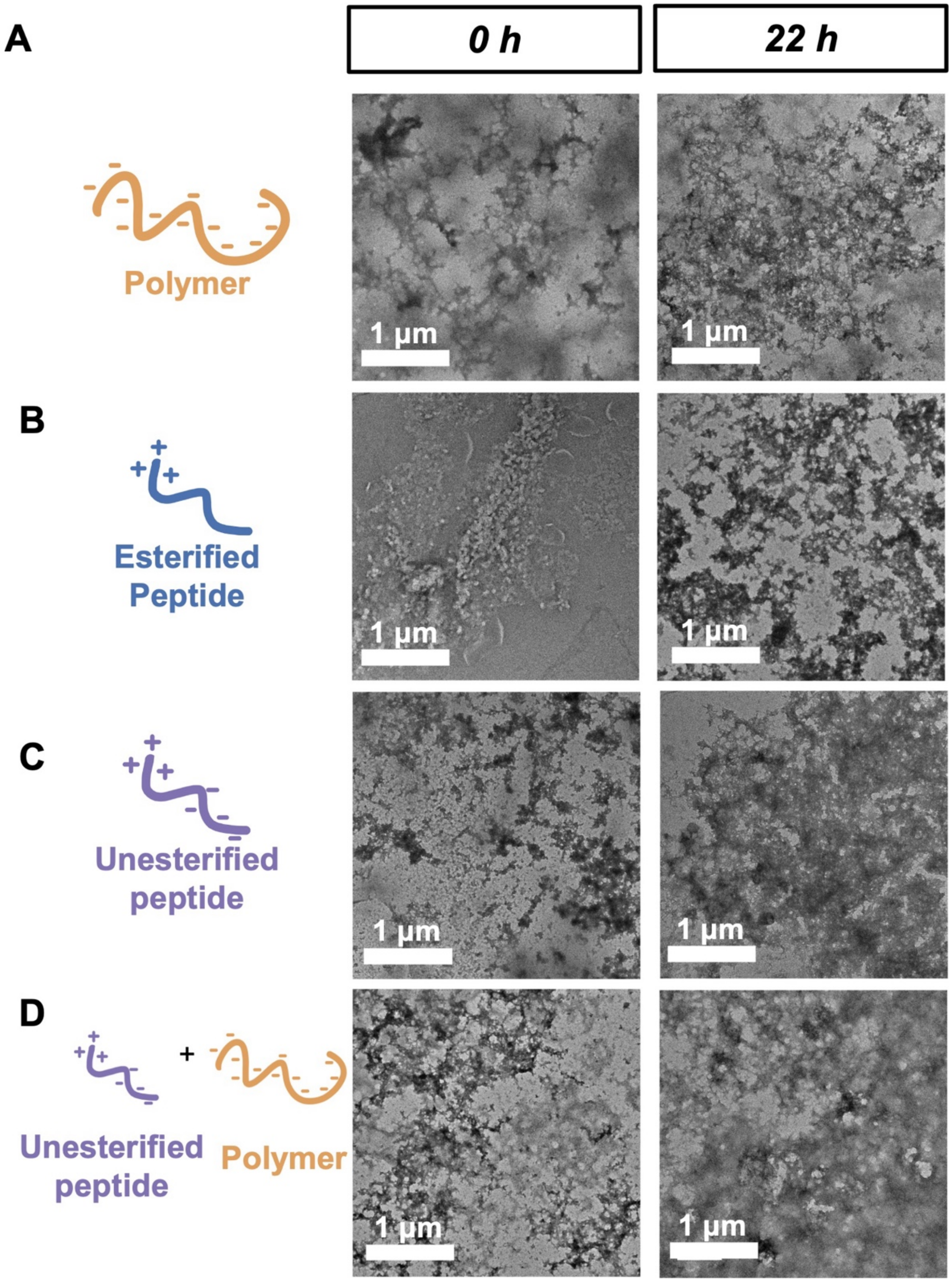

Figure S9. TEM of controls of: A) Polymer; B) Esterified peptide; C) Unesterified peptide; and D) Unesterified peptide + polymer; in 38 mM potassium phosphate buffer, at 0 and 22 h, showing no evidence of the network structures present in blends of polymer + esterified peptide.

Optical microscopy

We used optical microscopy to visualize changes to the complexes and controls occurring on the microscale. Images of the esterified peptide + polymer mixture (**Figure S10**) were compared to control mixtures of polymer, unesterified peptide, unesterified peptide + polymer, and esterified peptide (**Figure S11**) over 24 h. Initially (t = 0 h), the esterified peptide + polymer forms particles, consistent with the high turbidity at initial timepoints. Interestingly, these particles do not disappear by 24 h, which may be reasonable since the turbidity does not drop to baseline by 24 h, possibly owing to the presence of esterified variants still present (**Figure 2B**). To analyze the size distribution of these particles (**Figure S10**), which appeared more cylindrical than spherical, we calculated the area of the particles using the longest end-to-end distance of a particle as the length, and the shortest end-to-end distance as the width.

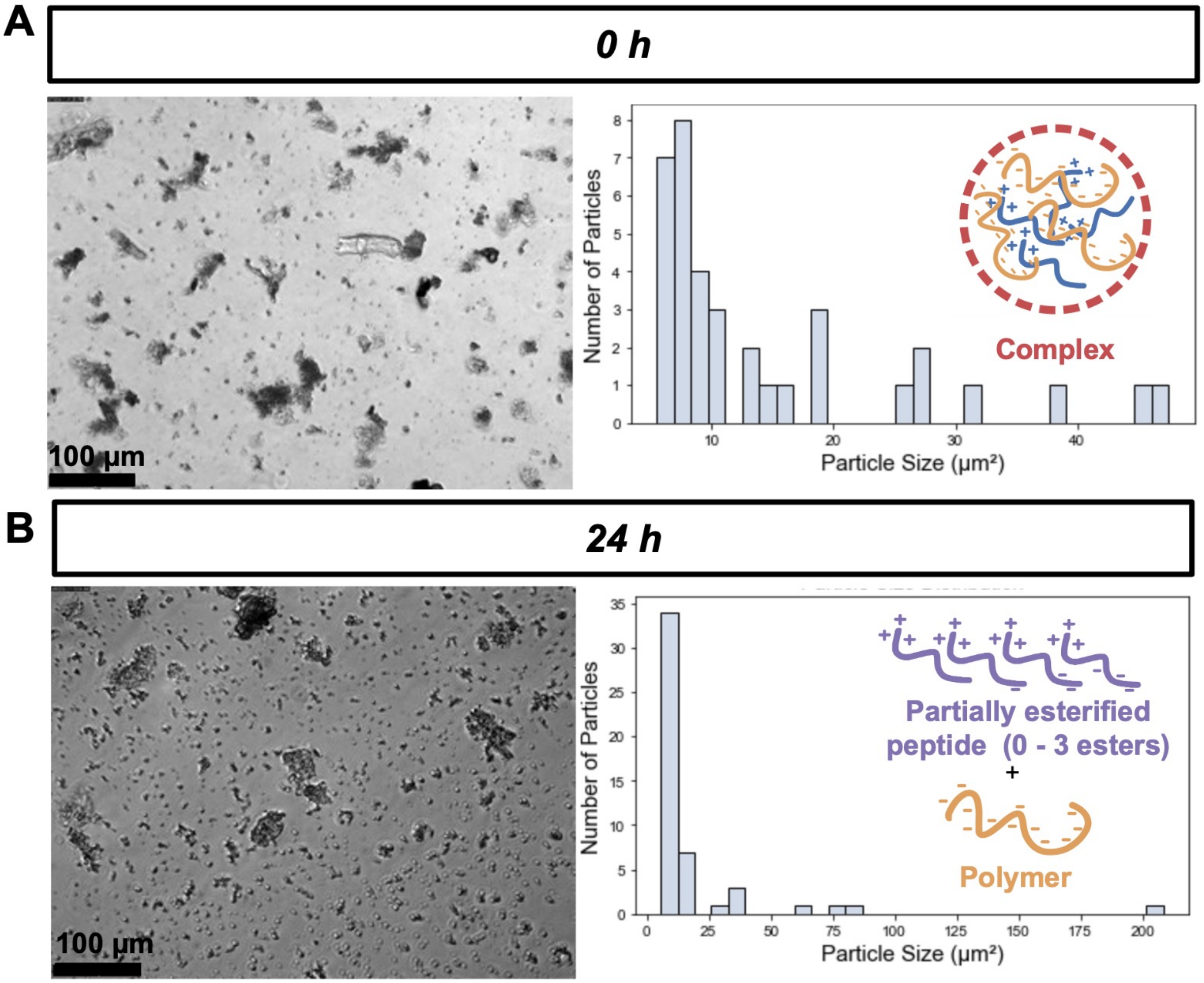

Figure S10. Optical microscopy of esterified peptide + polymer mixture shown after: A) 0 h; and B) 24 h; of incubation in 38 mM potassium phosphate buffer, during which fully esterified peptide hydrolyses into partially esterified peptide.

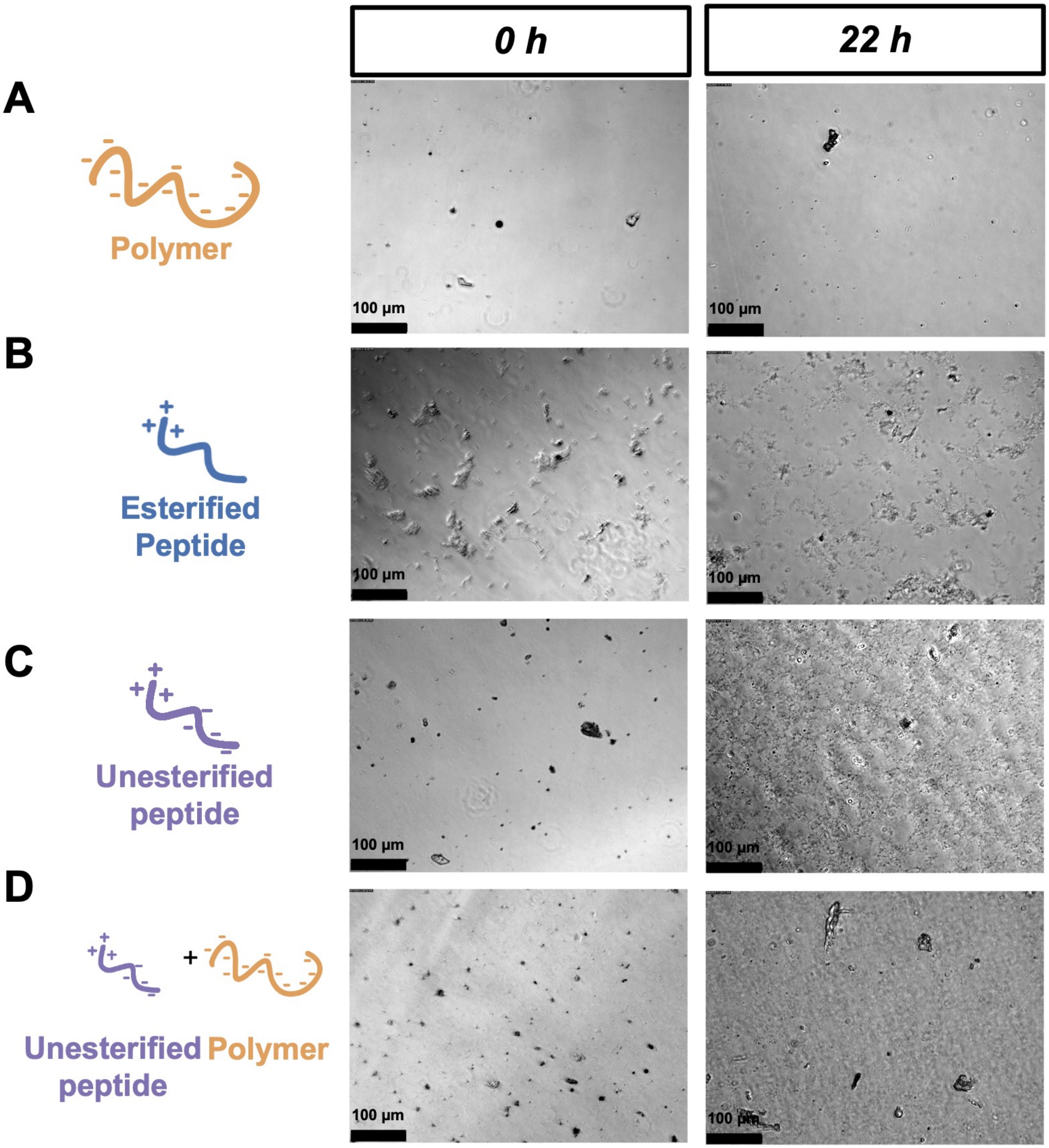

Figure S11. Optical microscopy of controls: A) Polymer; B) Esterified peptide; C) Unesterified peptide; and D) Unesterified peptide + polymer; in 38 mM potassium phosphate buffer, over 22 h.

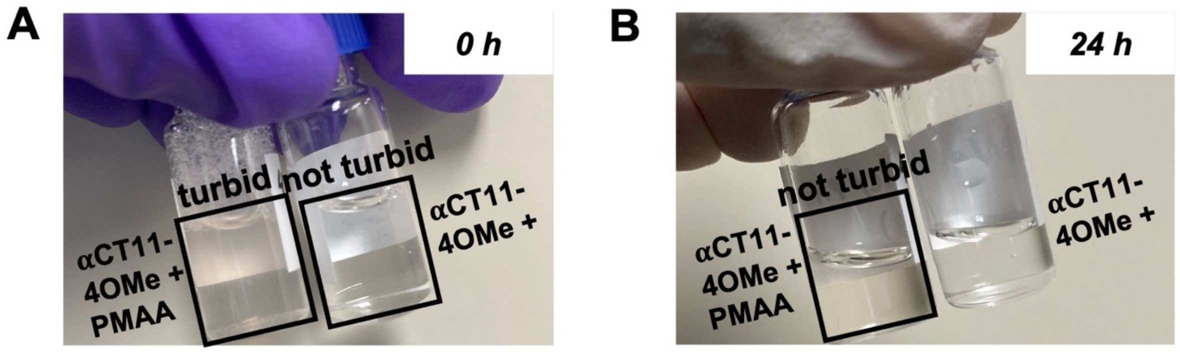

Figure S12: Representative images comparing esterified peptide + polymer mixture to esterified peptide control at: A) 0 h, where esterified peptide + polymer mixture (left) appears turbid, while esterified peptide control (right) does not, ruling out esterified peptide aggregation as a cause for turbidity; and at B) 24 h, where the esterified peptide + polymer mixture, loses turbidity, indicating complex dissociation.

Effects of gravitational settling on turbidity

Turbidity of esterified peptide + polymer (resuspended before measurement)

While our complexes decrease turbidity as they dissociate, we were concerned that as they start to settle out, light would have a clearer path resulting in a lower turbidity. This would convolute our ability to correlate complex dissociation to lower turbidity. To assess this, right before each reading, we gently pipetted the mixture in the well plate up and down, a few times, to resuspend any settled particles. The turbidity of the esterified peptide + polymer mixtures (**Figure S13**), with and without disruption, over 20 h, look very similar, suggesting that under these conditions, gravitational settling does not impact turbidity over time.

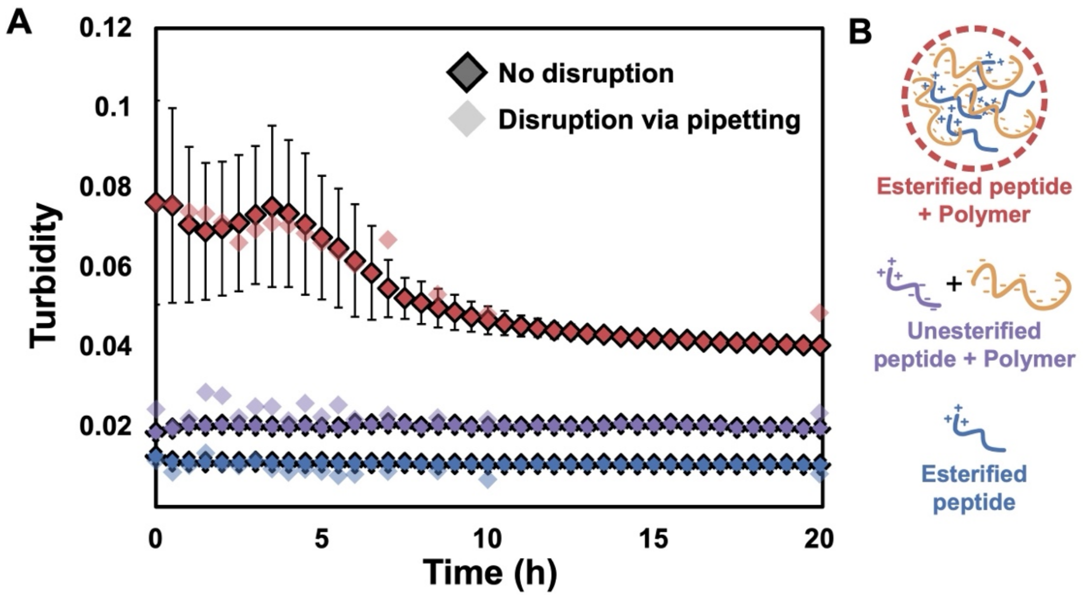

Figure S13. Effects of gravitational settling of complexes on turbidity: A) Comparing turbidity before disruption (opaque traces) and after disruption (transparent traces); and B) Schematic of control groups used for this turbidity study.

Effect of peptide, polymer and salt concentrations, buffer type, pH correction and polymer length on turbidity

Turbidity of esterified peptide + polymer in MOPS and HEPES buffer

To eliminate the possibility that turbidity of the esterified peptide + polymer mixture was a result of the divalent anionic potassium phosphate buffer (PB) cross linking the cationic esterified peptide, we repeated our turbidity experiments in 2 different zwitterionic buffers – MOPS and HEPES, at neutral pH. As seen in **Figure S14**, turbidity also occurs at neutral pH in MOPS and HEPES buffer, confirming that potassium phosphate buffer does not cross link the peptide, and that the esterified peptide + polymer mixture complex in all 3 buffers. The initial high turbidity in HEPES buffer is 3X that in PB buffer, and 2X that in MOPS buffer. Further, unlike in MOPS and PB where the peptide + polymer mixture turbidity returns to the level of the unesterified mixture after 24 h, in HEPES, the turbidity

remains higher. The extended turbidity in HEPES is consistent with slower ester hydrolysis measured by RP-HPLC in HEPES vs. PB.

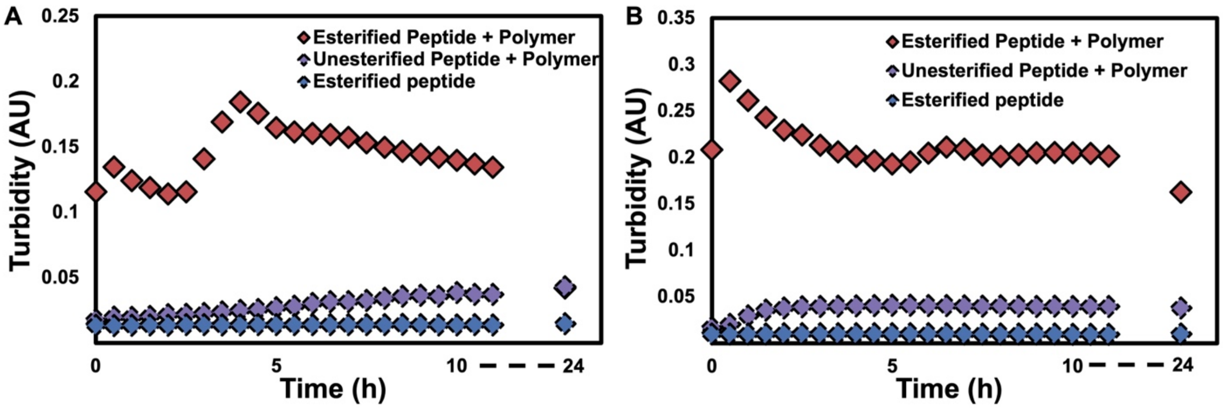

Figure S14. Turbidimetry to track complexation and dissociation of esterified peptide + polymer mixtures (0.5 mM polymer, 3.32 mM esterified peptide) in: A) 38 mM MOPS buffer; and B) 38 mM HEPES buffer; over 24 h. Note that the 24 h point in MOPS buffer overlaps with the unesterified peptide + polymer mixture turbidity.

Turbidimetry of esterified peptide + polymer complexes in 2X buffer (76 mM)

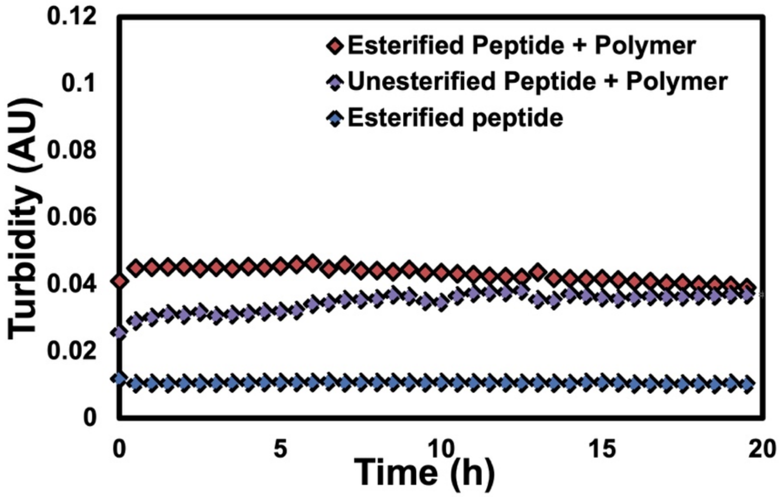

Figure S15. Turbidimetry to track complexation and dissociation of esterified peptide + polymer mixtures (0.5 mM polymer, 3.32 mM esterified peptide) in 76 mM (2X salt concentration) over 20 h.

Turbidimetry of esterified peptide + polymer complexes across different peptide-polymer concentrations

After initial experiments showing reversible complexation, we tested different concentrations of esterified peptide-polymer to find a suitable concentration to conduct our following studies for 3 main reasons: 1) understanding the effects of concentration on complexation, 2) finding a concentration where the complex is dispersed and not precipitated (to get more reliable turbidimetry readings) and 3) conserving material where possible. We repeated turbidity measurements at 4 different concentrations as shown below, where the peptide was scaled to maintain a charge ratio of 1:1 with the polymer. Decreasing the concentration decreased turbidity (**Figure S16**), and the esterified peptide + polymer mixtures consistently showed a turbidity higher than the controls. All subsequent studies were conducted at a concentration of 0.5 mM polymer (and 3.32 mM peptide) to get good separation between the controls and the complexes, alongside preventing precipitation.

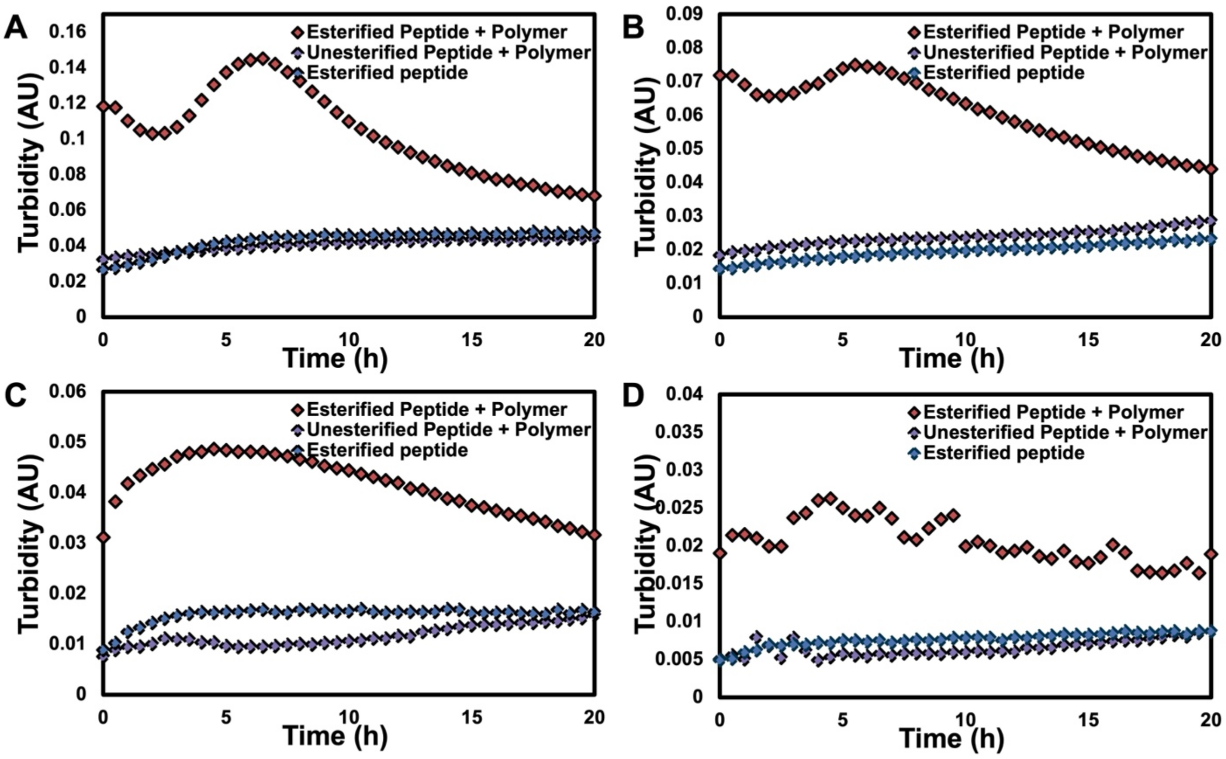

Figure S16. Turbidimetry to track complexation and dissociation of esterified peptide + polymer mixtures at: A) 0.75 mM polymer, 4.98 mM esterified peptide, 57 mM salt; B) 0.5 mM polymer, 3.32 mM esterified peptide, 38 mM salt; C) 0.25 mM polymer, 1.66 mM esterified peptide, 19 mM salt; and D) 0.125 mM polymer, 0.83 mM esterified peptide, 9.5 mM salt; over 20 h. Note that the esterified peptide concentrations were calculated based on the polymer concentrations to yield a 1:1 charge ratio at neutral pH.

Turbidimetry of esterified peptide + polymer complexes without pH correction

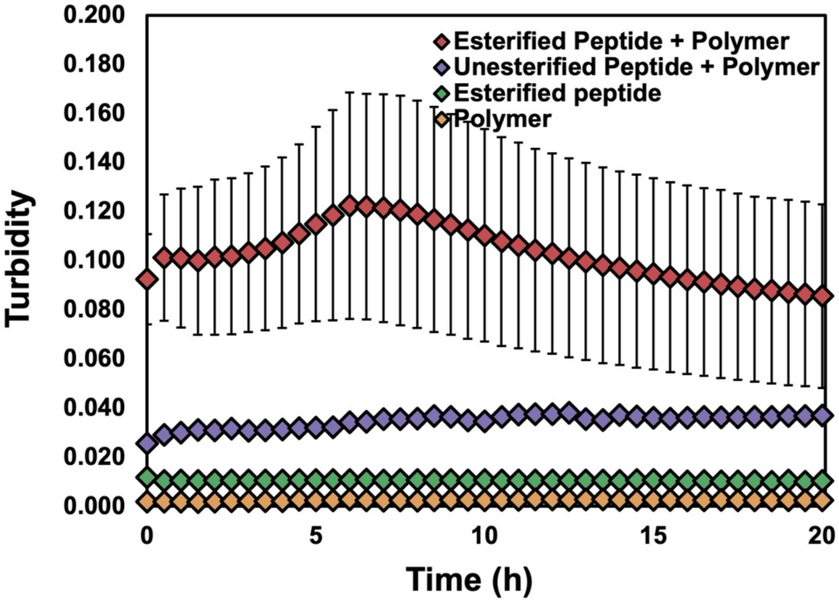

Figure S17. Turbidimetry to track complexation and dissociation of esterified peptide + polymer mixtures (0.5 mM polymer, 3.32 mM esterified peptide) in 38 mM potassium phosphate buffer, without pH correction, over 20 h.

Turbidimetry of esterified peptide + polymer complexes with PMAA32 (DP = 32)

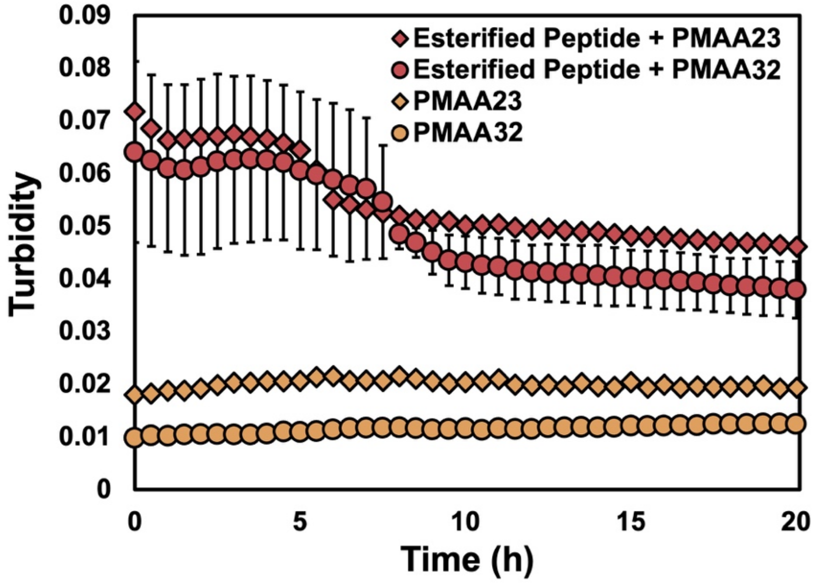

Figure S18. Turbidimetry to track complexation and dissociation of esterified peptide + polymer mixtures (0.3 mM polymer, 3.32 mM esterified peptide) in 38 mM potassium phosphate buffer, without pH correction, over 20 h.

Effects of aspartimide intermediates on turbidity

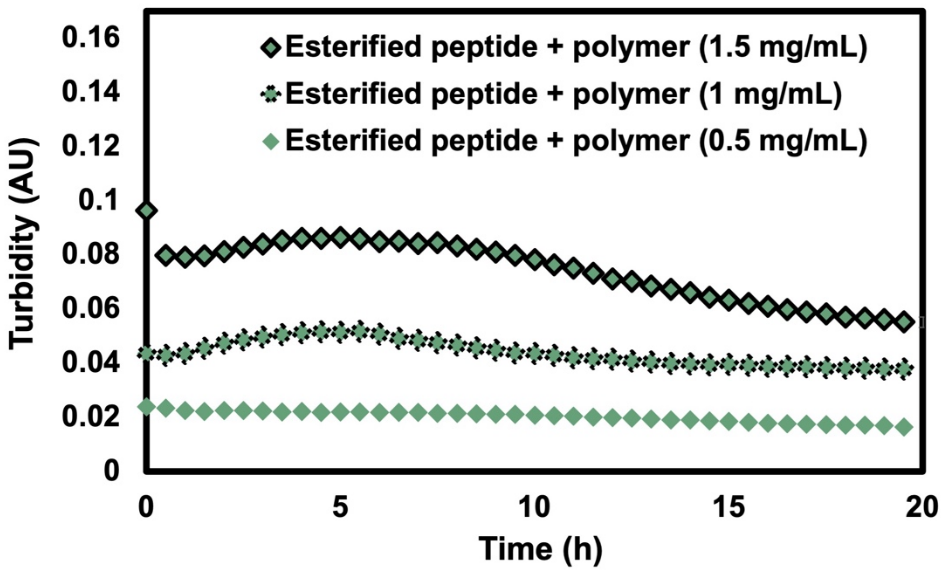

Figure S19. Turbidimetry to track complexation and dissociation of esterified peptide (RPRPEELEI-4OMe) + polymer mixtures at: 0.75 mM polymer (with 4.98 mM esterified peptide, 57 mM salt); 0.5 mM polymer (with 3.32 mM esterified peptide, 38 mM salt); and 0.25 mM polymer (with 1.66 mM esterified peptide, 19 mM salt), over 20 h, in pH 10 carbonate buffer. The concentrations of esterified peptide and buffer were scaled by the same amount as the polymer concentration.

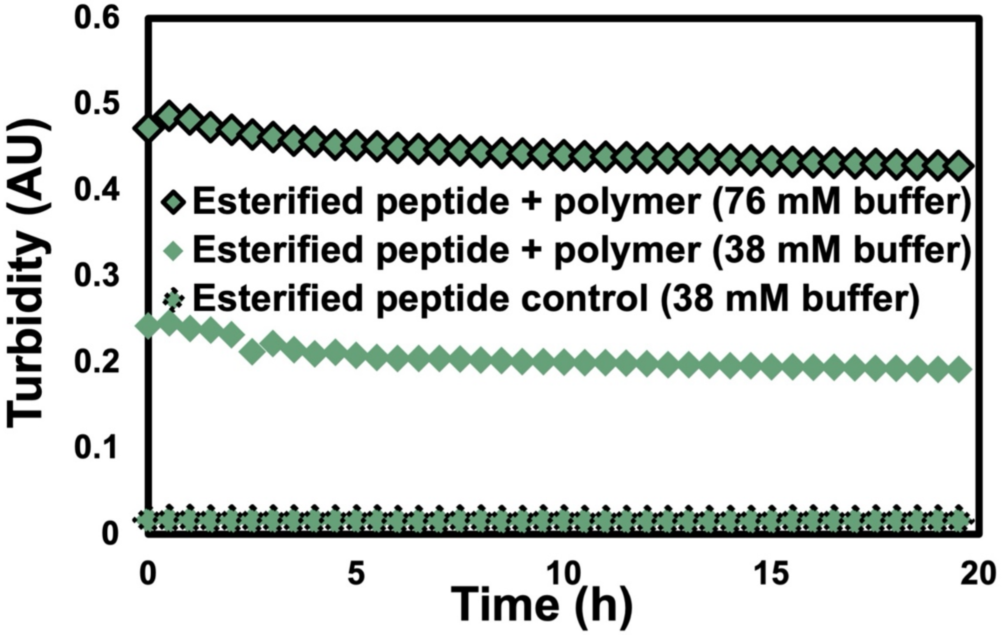

Figure S20. Turbidimetry to track complexation and dissociation of esterified peptide (RPRPEELEI-4OMe) + polymer mixtures at 38 mM salt concentration (1X) vs. 76 mM salt concentration (2X), over 20 h

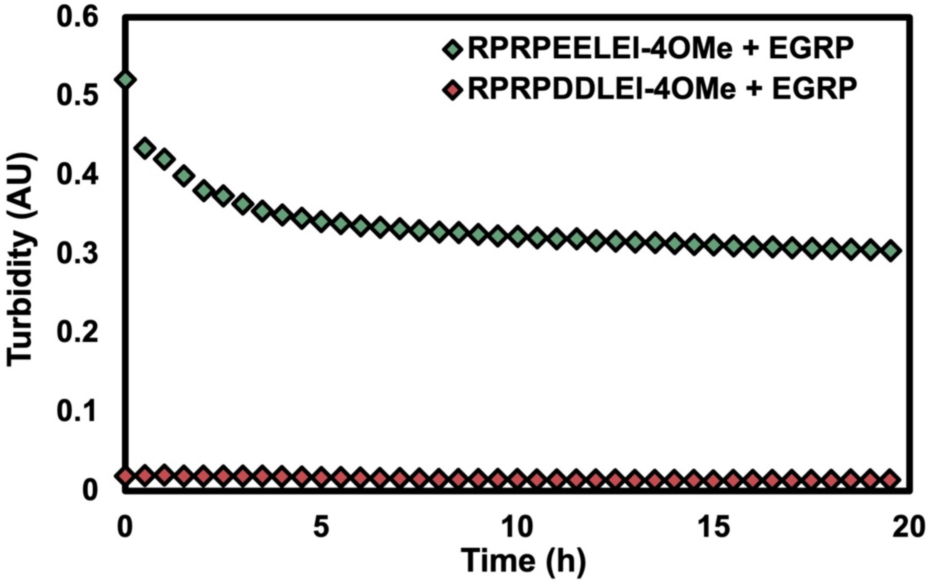

Figure S21. Turbidimetry to compare complexation and dissociation of end-group replaced polymer (EGRP) with RPRPEELEI-4OMe (green) and RPRPDDLEI-4OMe (pink), at 38 mM salt concentration, over 20 h.

Tracking hydrolysis during reversible complexation

Analytical reverse-phase high-performance liquid-chromatography (RP-HPLC) of esterified peptide under different conditions

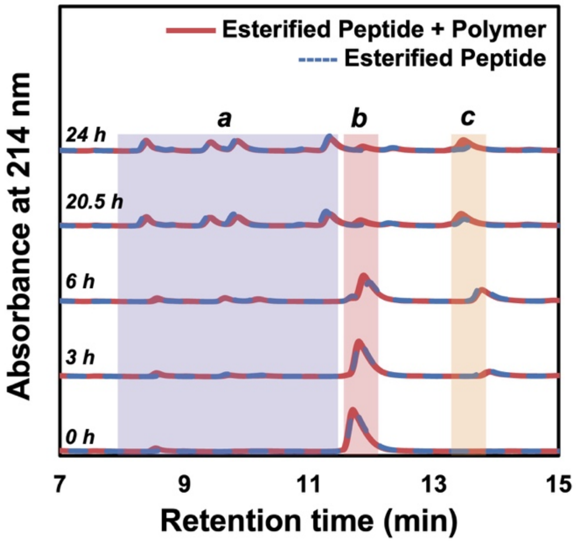

Figure S22. Analytical RP-HPLC tracking hydrolysis of esterified peptide (RPRPDDLEI-4OMe) in the presence (pink, 3 replicates) and absence (blue, dashed) of polymer in 38 mM potassium phosphate buffer (pH 7.35-7.4) over 24 h. Peaks shown in region a shows hydrolysis intermediates of esterified peptide containing 1-3 esters, peak b shows fully esterified peptide and peak c shows a more hydrophobic hydrolysis intermediate.

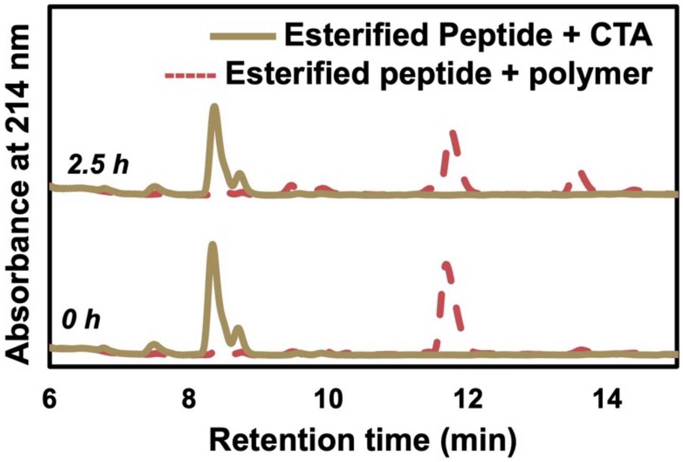

Figure S23. RP-HPLC tracking hydrolysis of esterified peptide (RPRPDDLEI-4OMe) in the presence of chain transfer agent (brown) and presence of polymer (pink, dashed) in 38 mM potassium phosphate buffer (pH 7.35-7.4) over 2.5 h.

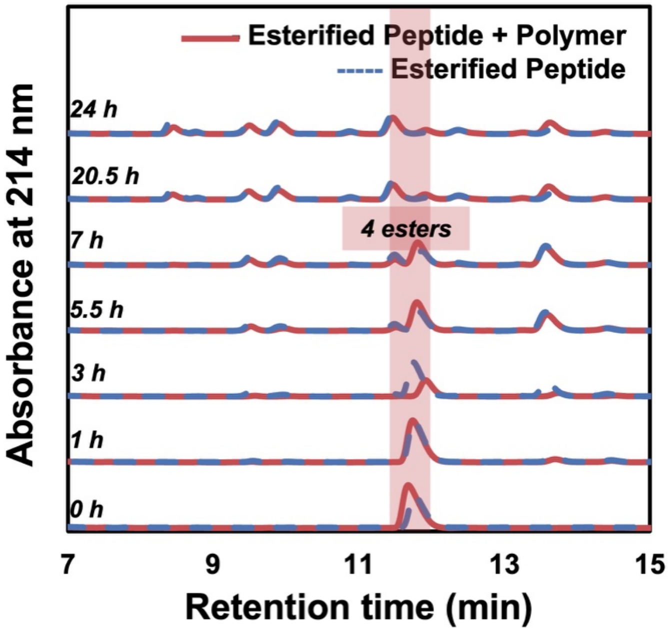

Figure S24. RP-HPLC tracking hydrolysis of esterified peptide (RPRPDDLEI-4OMe) in the presence (pink) and absence (blue, dashed) of polymer over 24 h, in 76 mM potassium phosphate buffer.

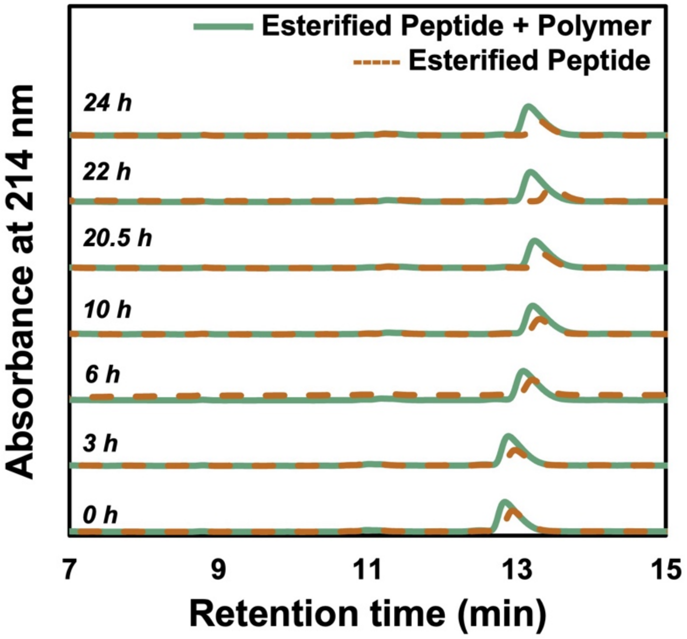

Figure S25. RP-HPLC tracking hydrolysis of esterified peptide (RPRPEELEI-4OMe, no aspartimides) in the presence (green) and absence (orange, dashed) of polymer over 24 h, in 38 mM potassium phosphate buffer (pH 7.35-7.4)

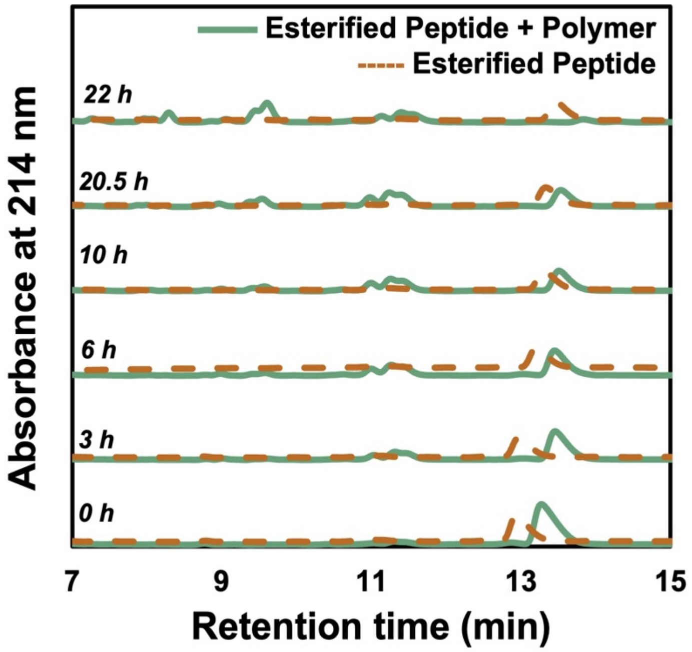

Figure S26. RP-HPLC tracking hydrolysis of esterified peptide (RPRPEELEI-4OMe, no aspartimides) in the presence (green) and absence (orange, dashed) of polymer over 22 h, in 38 mM carbonate buffer (pH 10).

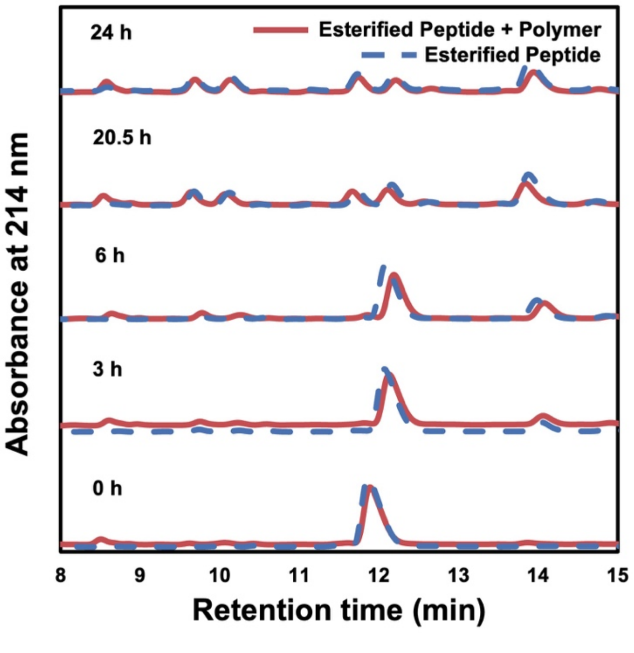

Figure S27. RP-HPLC tracking hydrolysis of esterified peptide (RPRPDDLEI-4OMe) in the presence (pink) and absence (blue, dashed) of polymer over 24 h, in 38 mM MOPS buffer (pH 7.4).

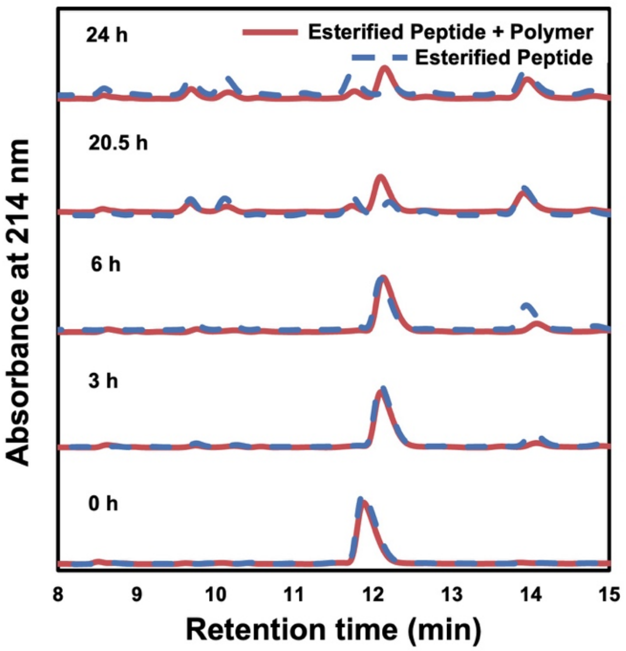

Figure S28. RP-HPLC tracking hydrolysis of esterified peptide (RPRPDDLEI-4OMe) in the presence (pink) and absence (blue, dashed) of polymer over 24 h, in 38 mM HEPES buffer (pH 7.4).

Liquid chromatography quadrupole time of flight (LC-QTOF) mass spectrometry of RPRPDDLEI-4OMe + polymer complexes

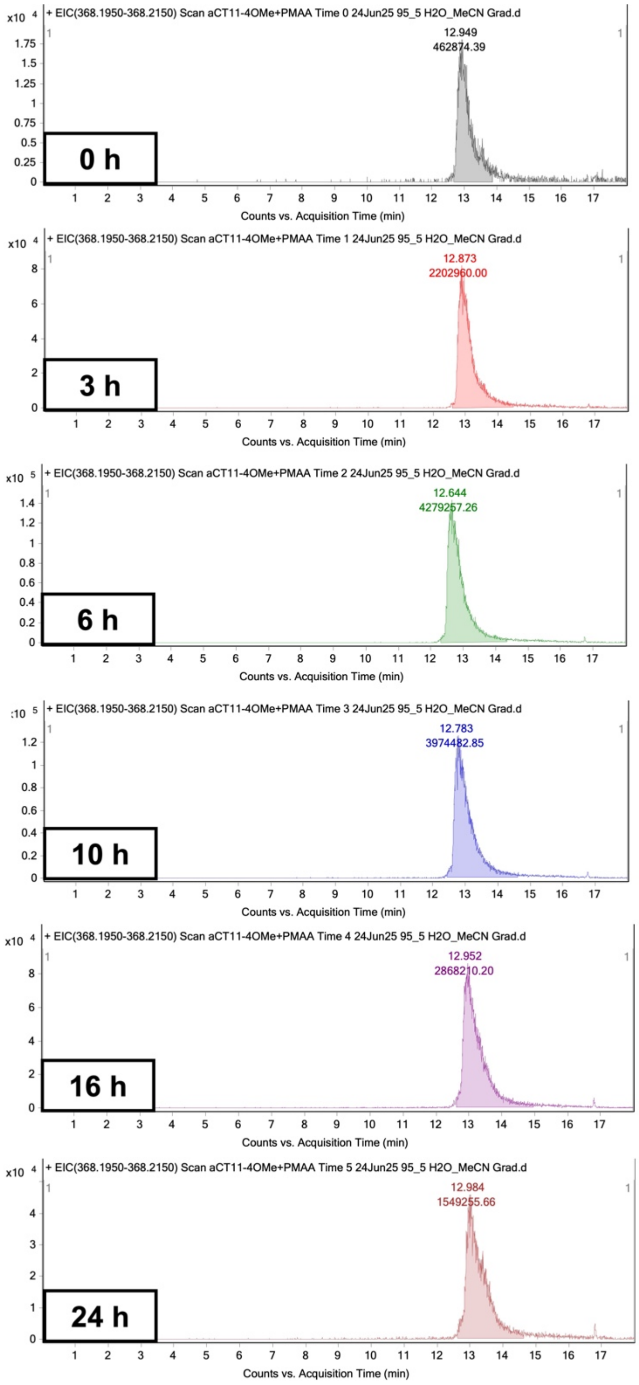

Figure S29. LC-QTOF EICs extracting αCT11-2OMe-2imide peak area over 24 h

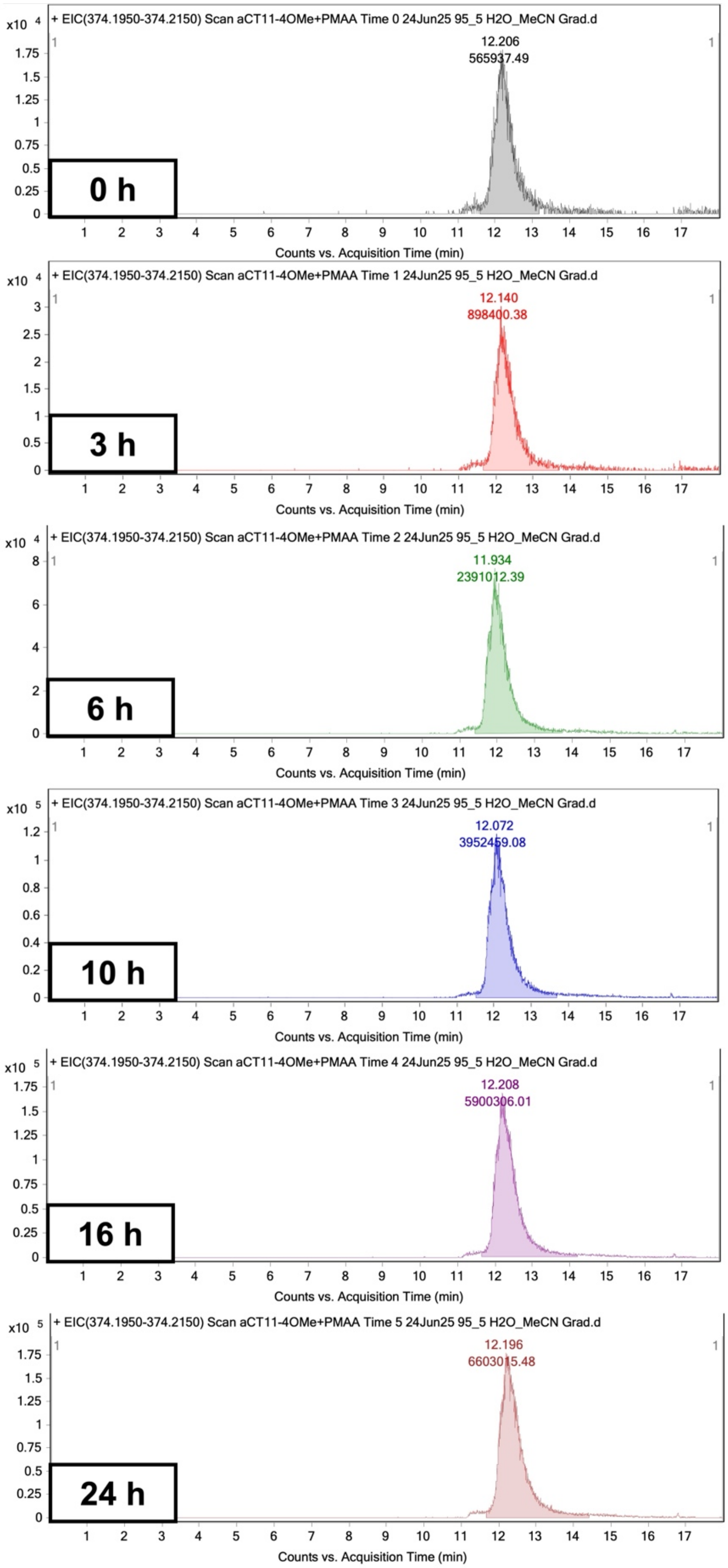

Figure S30. LC-QTOF EICs extracting **α**CT11-2OMe-1imide peak area over 24 h

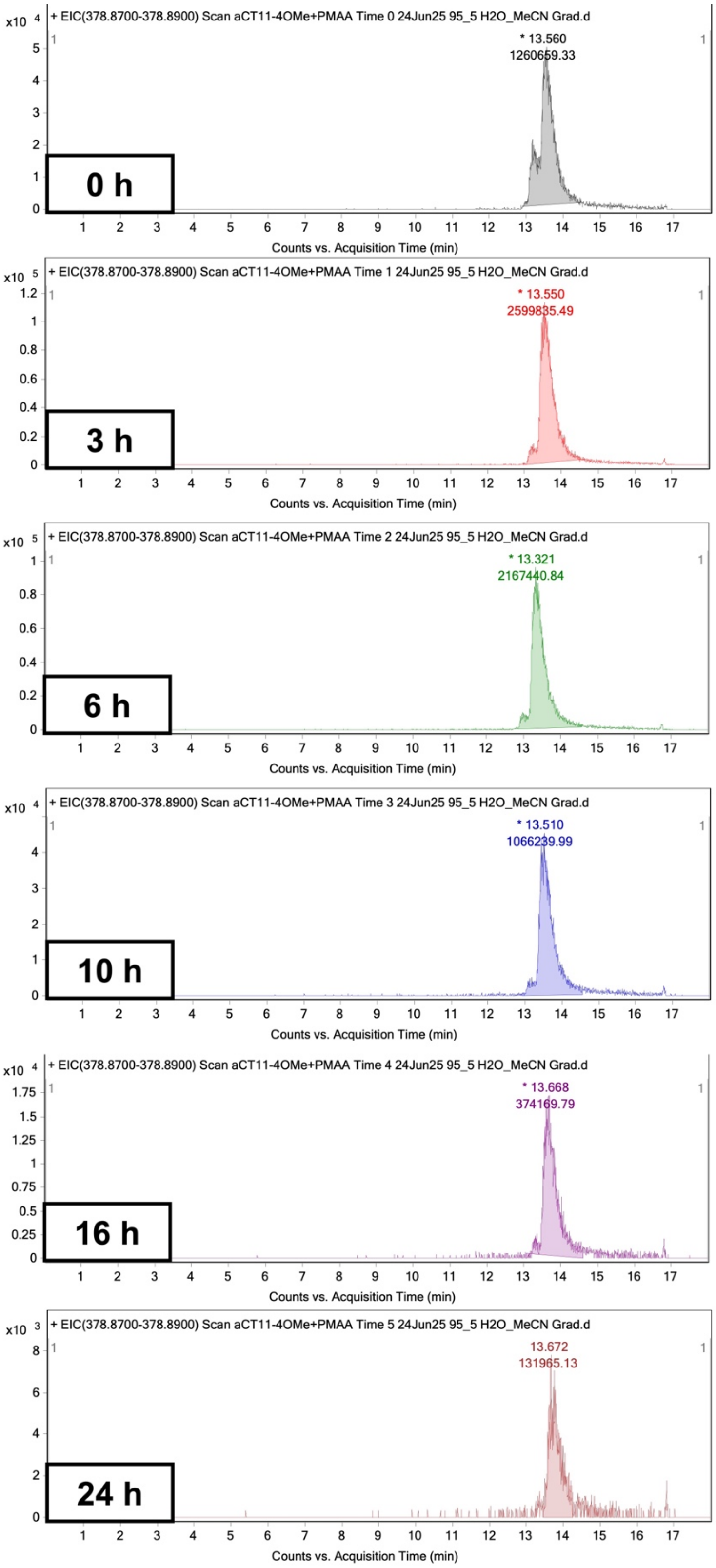

Figure S31. LC-QTOF EICs extracting **α**CT11-3OMe-1imide peak area over 24 h

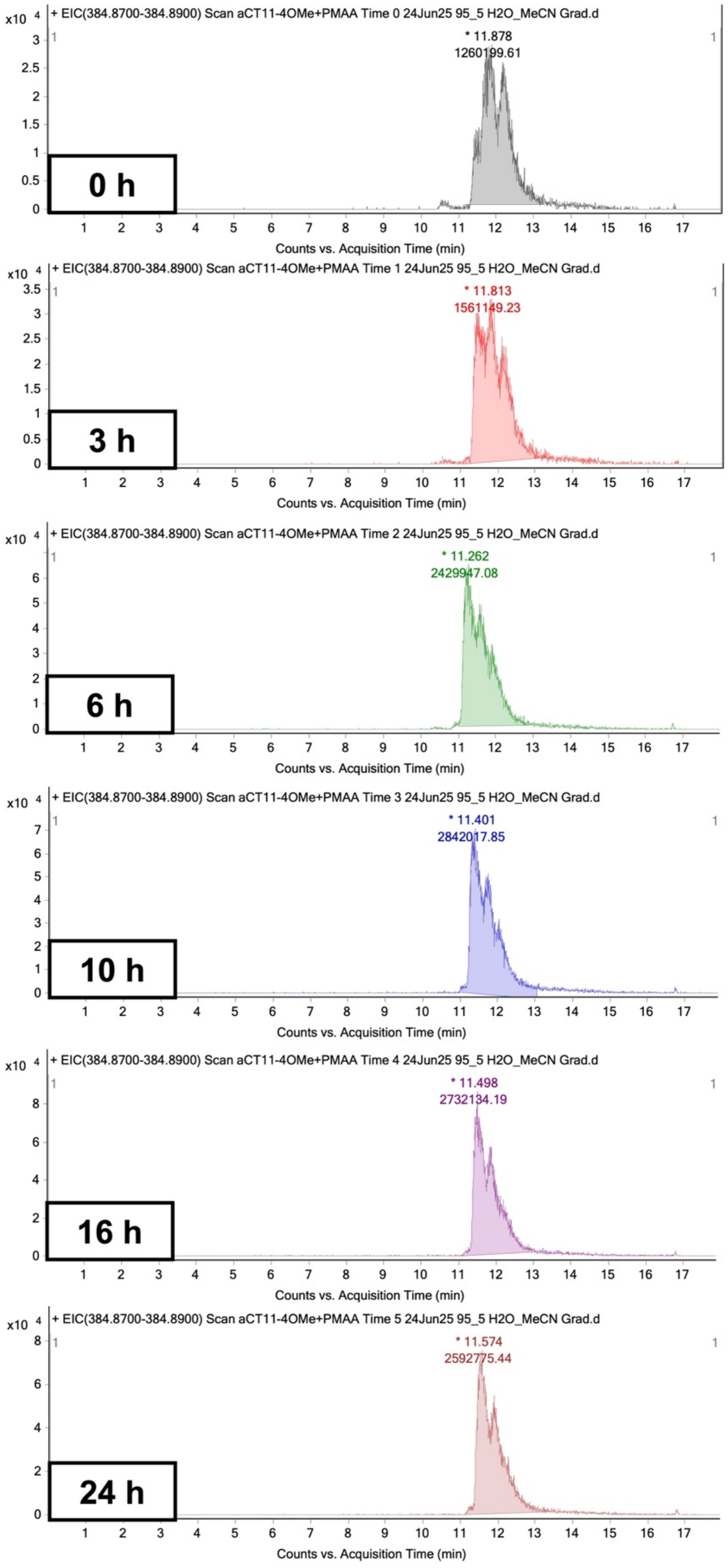

Figure S32. LC-QTOF EICs extracting **α**CT11-3OMe peak area over 24 h

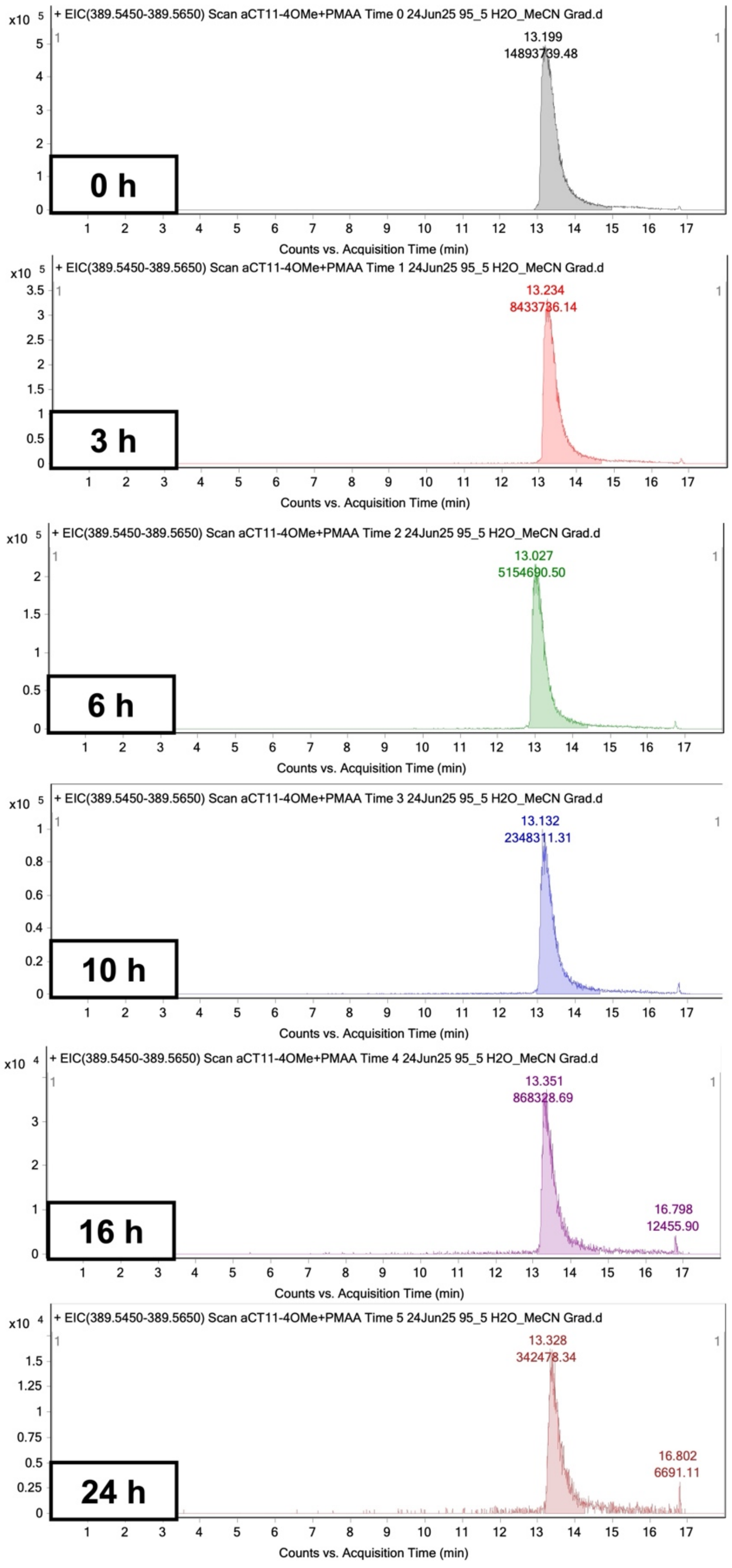

Figure S33. LC-QTOF EICs extracting **α**CT11-4OMe peak area over 24 h

Liquid chromatography quadrupole time of flight (LC-QTOF) mass spectrometry of RPRPEELEI-4OMe + polymer complexes

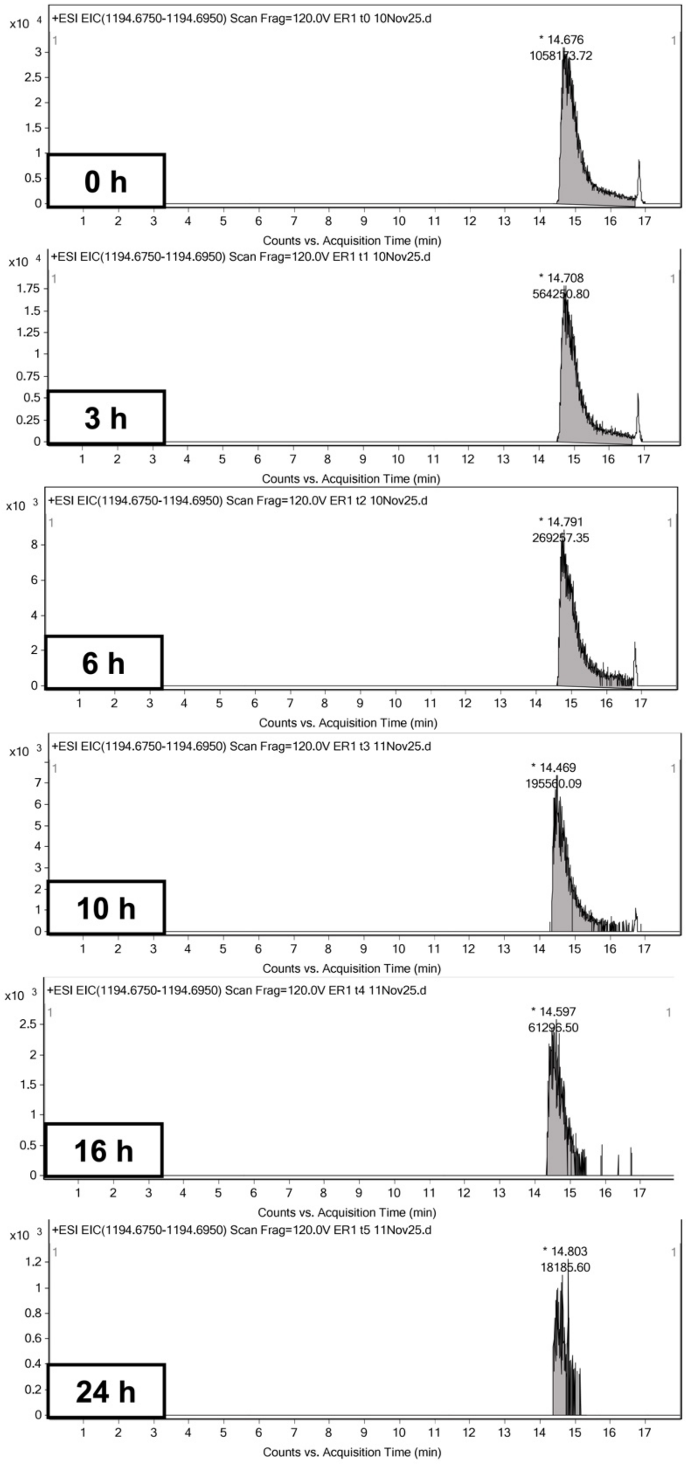

Figure S34. LC-QTOF EICs extracting RPRPEELEI-4OMe peak area over 24 h

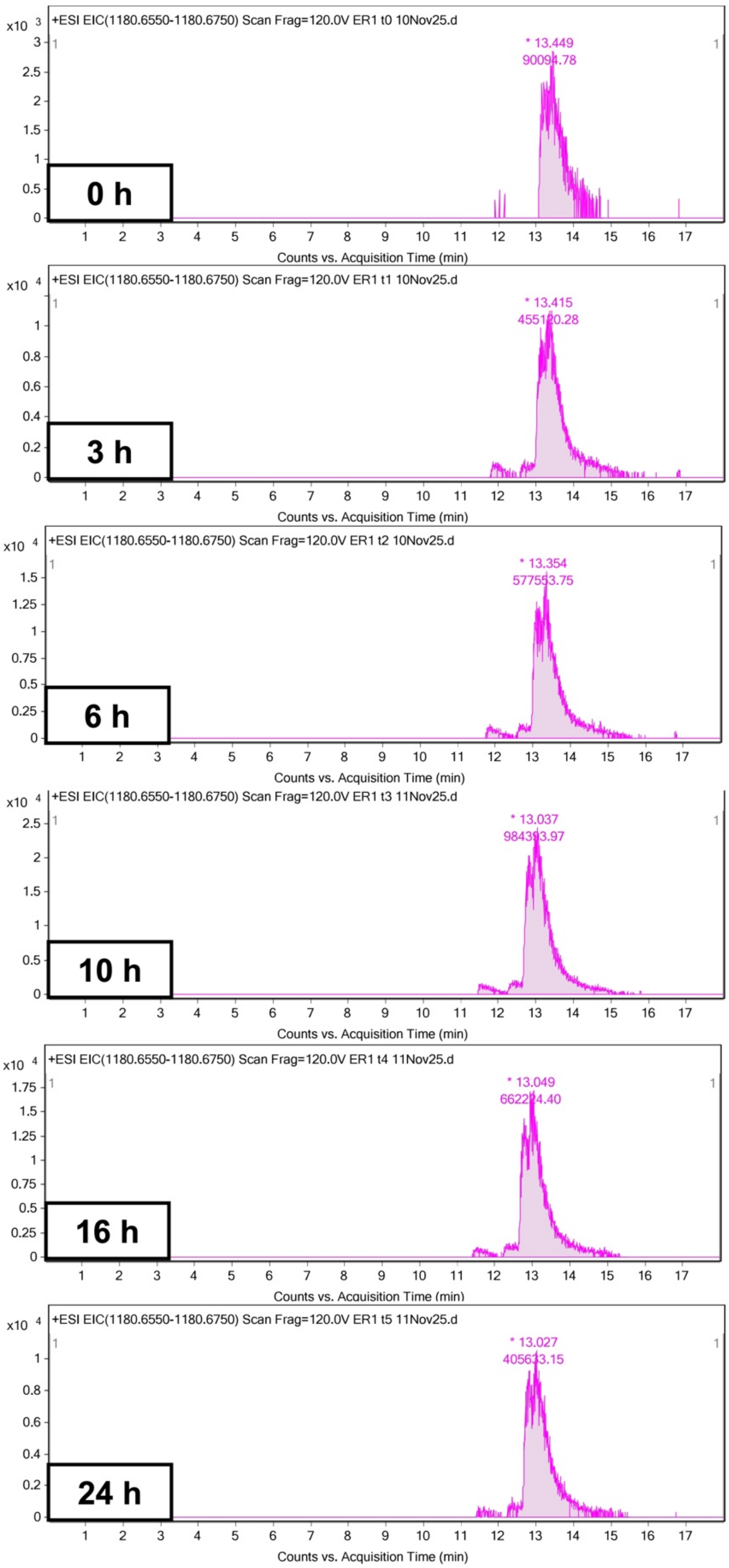

Figure S35. LC-QTOF EICs extracting RPRPEELEI-3OMe peak area over 24 h

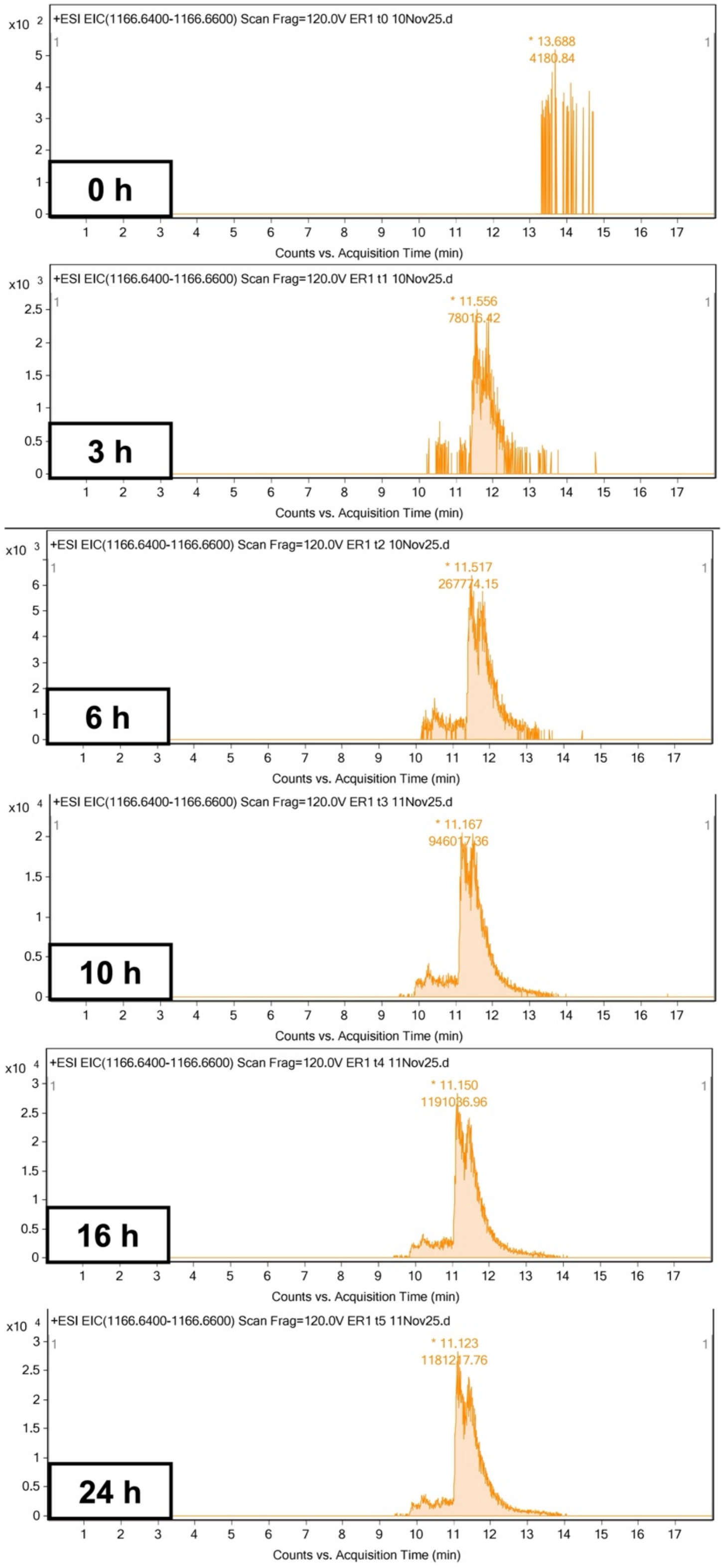

Figure S36. LC-QTOF EICs extracting RPRPEELEI-2OMe peak area over 24 h

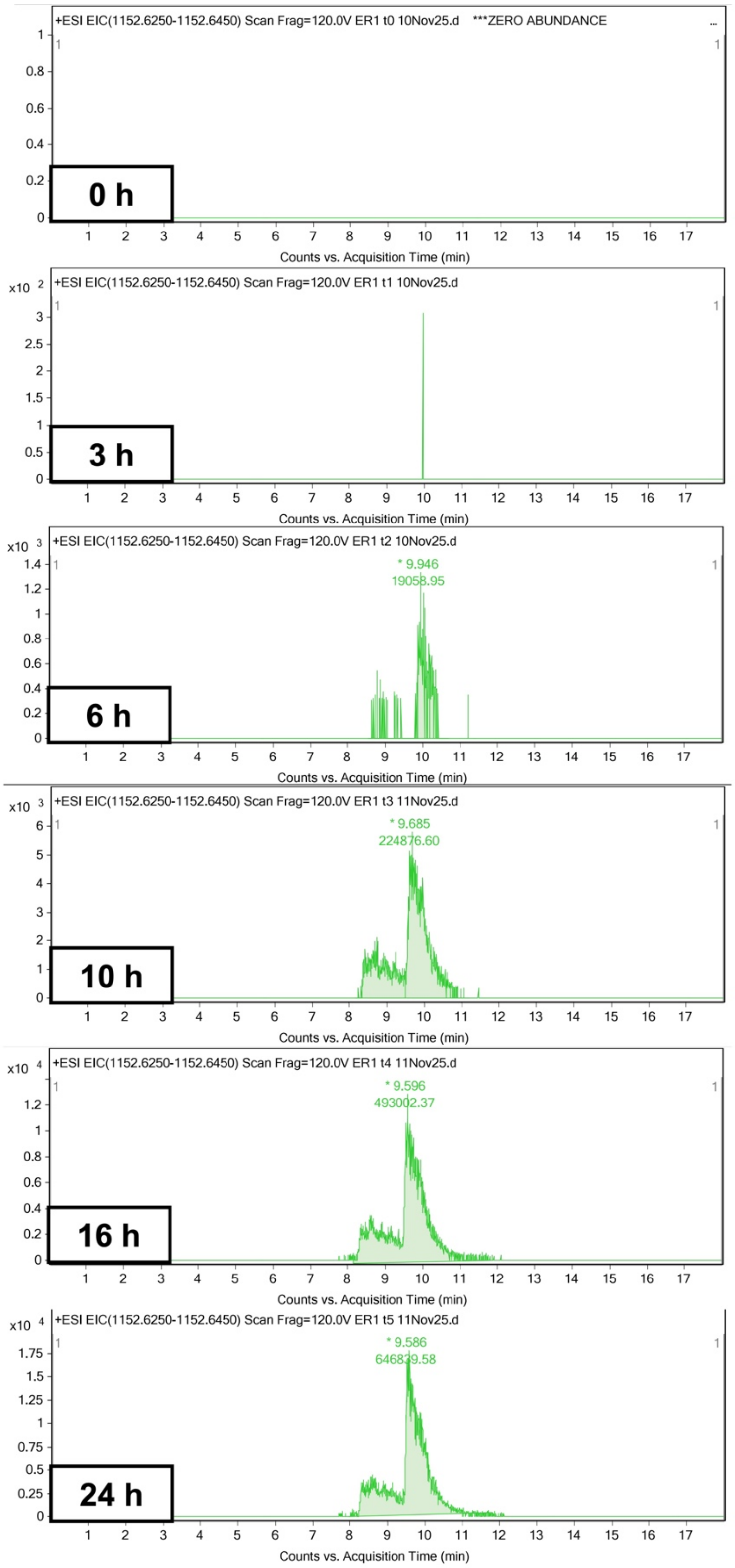

Figure S37. LC-QTOF EICs extracting RPRPEELEI-1OMe peak area over 24 h

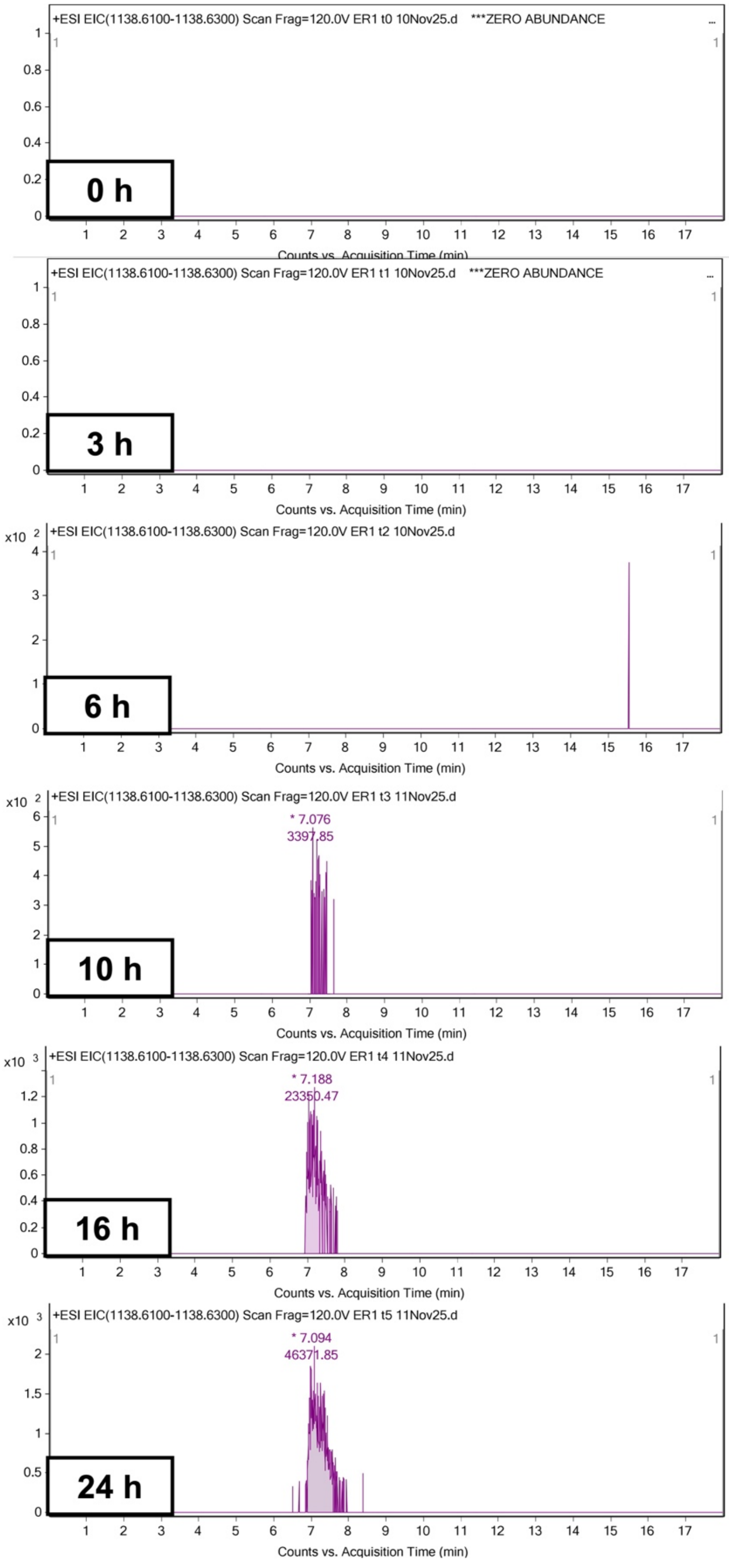

Figure S38. LC-QTOF EICs extracting RPRPEELEI peak area over 24 h

Monitoring pH of esterified peptide + polymer and controls over 20 h

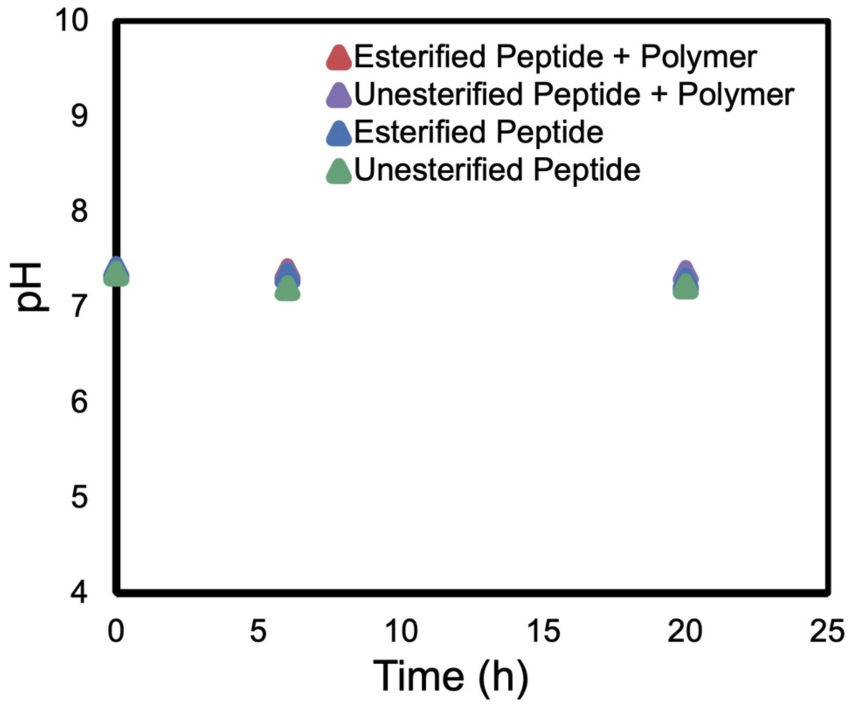

Figure S39: pH monitoring of esterified peptide + polymer mixture (red, 3 replicates), unesterified peptide + polymer (purple), esterified peptide (blue) and unesterified peptide (green), over 20 h.
